# Hepatobiliary phase imaging in HCC: revisiting diagnostic algorithms and clinical utility of gadoxetic acid-enhanced MRI across international guidelines

**DOI:** 10.1186/s13244-026-02402-5

**Published:** 2026-09-28

**Authors:** Shuwei Zhou, Jie Yang, Shuhang Zhang, Yu Song, Shuang Li, Xiaofeng Yu, Siyu Wang, Shenghong Ju, Tian You, Yuan-Cheng Wang

**Affiliations:** 1 Department of Radiology, Zhongda Hospital, Nurturing Center of Jiangsu Province for State Laboratory of AI Imaging & Interventional Radiology, School of Medicine, Southeast University, Nanjing, China; 2 Department of Emergency Surgery, Kunshan Hospital of Chinese Medicine, Kunshan, China; 3 Department of Radiology, The First Hospital of Hunan University of Chinese Medicine, Changsha, China

**Keywords:** Carcinoma (Hepatocellular), Diagnosis, Gadoxetic acid, Guideline, Magnetic resonance imaging

## Abstract

**Abstract:**

Hepatocellular carcinoma (HCC) remains a major cause of cancer-related mortality worldwide. In at-risk populations, noninvasive imaging diagnosis relies primarily on the vascular hallmarks of arterial phase hyperenhancement and washout appearance. However, these features are often absent in early hepatocarcinogenesis, thereby limiting timely detection. Gadoxetic acid-enhanced MRI has expanded the role of imaging in HCC, yet its clinical value is often oversimplified as a comparison of diagnostic performance with CT or extracellular contrast MRI. A critical issue is that the same imaging feature, particularly hepatobiliary phase (HBP) hypointensity, carries different diagnostic consequences across international frameworks. Because HBP signal loss reflects early hepatocellular dysfunction that may precede typical vascular hallmarks, gadoxetic acid-enhanced MRI provides a biologically driven sensitivity advantage for detecting small and early-stage HCC. However, its apparent performance varies largely according to diagnostic intent: sensitivity-oriented Asian guidelines incorporate HBP hypointensity as a major feature to enable earlier detection, whereas Western guidelines prioritize specificity in high-stakes decision contexts, such as liver transplantation, and therefore restrict its diagnostic weight. This intent-dependent interpretation explains much of the inconsistency reported in the literature and underscores the limitations of pooled, guideline-agnostic performance estimates. Beyond diagnosis, HBP imaging also offers complementary information for assessing treatment response, although it remains underutilized in current criteria. Overall, the clinical value of gadoxetic acid-enhanced MRI lies not in universal superiority but in context-aligned application, shifting the focus from modality comparison to optimization according to downstream clinical consequences.

**Key Points:**

- **Question** Gadoxetic acid-enhanced MRI is interpreted differently across international hepatocellular carcinoma diagnostic guidelines, resulting in uncertainty regarding optimal imaging criteria and clinical decision-making.
- **Findings** Hepatobiliary phase hypointensity reflects early hepatocellular dysfunction and improves sensitivity for small and early-stage HCC, but its impact varies according to diagnostic intent.
- **Critical Relevance Statement** An intent-aligned interpretation strategy may help reconcile guideline heterogeneity and optimize the use of gadoxetic acid-enhanced MRI, particularly in the evaluation of small HCC.

**Graphical Abstract:**

**Figure:**
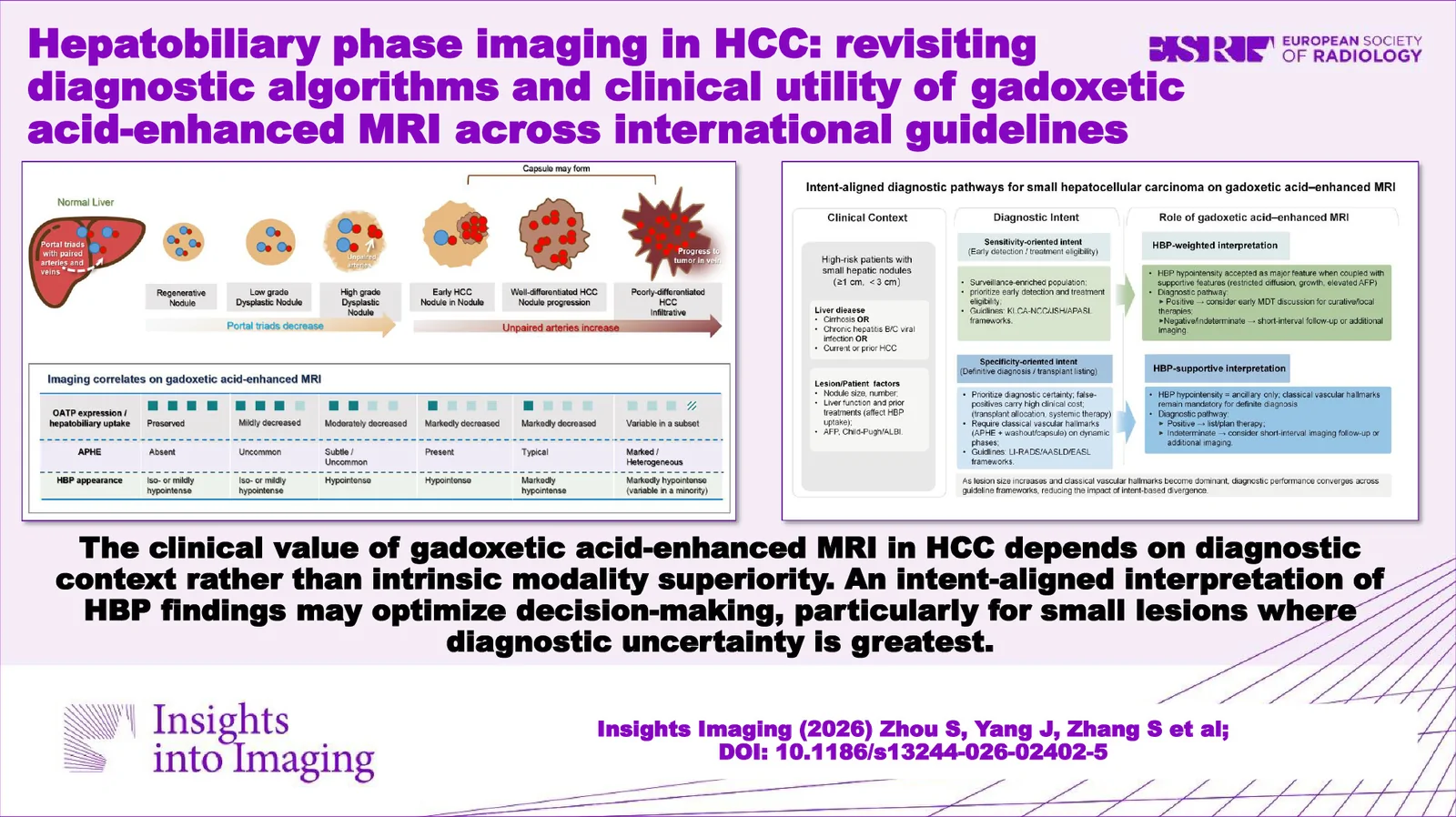

## Introduction

Hepatocellular carcinoma (HCC) is the third leading cause of cancer-related mortality worldwide [1, 2]. In high-risk patients, noninvasive diagnosis can be established using characteristic imaging hallmarks, most notably arterial phase hyperenhancement (APHE) followed by washout appearance on contrast-enhanced CT (CE-CT) or MRI. However, these vascular patterns are frequently absent in early or small HCCs, as such lesions often lack typical hemodynamic changes during the initial stages of hepatocarcinogenesis.

The advent of hepatocyte-specific contrast agents, particularly gadoxetic acid disodium, has markedly advanced the imaging evaluation of HCC. Approximately 50% of the injected dose is taken up by functioning hepatocytes and excreted via the biliary system, allowing hepatobiliary phase (HBP) imaging approximately 20 min post-injection. In contrast, most HCCs show reduced or absent uptake, thereby yielding high lesion-to-liver contrast. These properties provide gadoxetic acid-enhanced MRI with a distinct diagnostic advantage, particularly for small or atypical HCCs [3]. Nevertheless, its diagnostic performance is influenced not only by reduced arterial phase image quality, but also by the diagnostic criteria applied.

Substantial heterogeneity exists across international guidelines for the diagnosis and management of HCC. Asian guidelines frequently regard HBP hypointensity as a major diagnostic feature [4–6], whereas Western guidelines restrict it to an ancillary role, prioritizing specificity over sensitivity. Consequently, reported diagnostic performance of gadoxetic acid-enhanced MRI varies considerably across studies [7–9]. Importantly, most prior reviews and meta-analyses have focused on pooled diagnostic performance without adequately considering these differences in diagnostic frameworks [10–12]. Such an approach risks obscuring the true clinical value of gadoxetic acid-enhanced MRI, as identical imaging findings may yield markedly different sensitivity and specificity depending on the applied criteria.

This review systematically evaluates the role of gadoxetic acid-enhanced MRI in HCC diagnosis across major international guidelines, with particular emphasis on guideline-based diagnostic accuracy by lesion size, as well as its role in treatment response assessment.

## Materials and methods

This review was conducted based on a comprehensive literature search of PubMed from inception up to June 2025. The search was initially performed in January 2025 and updated in June 2025 using keyword combinations related to HCC, magnetic resonance imaging (MRI), contrast agents, clinical guidelines, and treatment response assessment. Only English-language publications were considered. The aim of the search strategy was to identify key original studies, review articles, international guidelines, and consensus statements relevant to imaging-based diagnosis and therapeutic decision-making in HCC. In addition, ongoing and recently completed clinical trials registered at ClinicalTrials.gov were reviewed when pertinent to emerging diagnostic or therapeutic concepts.

For diagnostic accuracy studies summarized in the tables, methodological quality was assessed using the Quality Assessment of Diagnostic Accuracy Studies-2 (QUADAS-2) tool. Risk of bias and applicability concerns were evaluated across the standard domains of patient selection, index test, reference standard, and flow and timing. The results of this quality assessment are summarized in Supplementary Fig. 1. The search strategy is described in Table 1.

**Table 1:** The search strategy summary

Items	Specification
Date of search	January 2025 and June 2025
Databases searched	PubMed
Search terms	“hepatocellular carcinoma”, “diagnosis”, “magnetic resonance imaging”, “MRI”, “gadoxetic acid”, “hepatobiliary contrast agent”, “extracellular contrast agent”, “guideline”, “LI-RADS”, “locoregional therapy”, “systemic therapy”, “immunotherapy”, “treatment response”
Timeframe	From inception to June 2025
Inclusion criteria	(1) Publications focusing on HCC, addressing imaging-based diagnosis, contrast agent–specific characteristics (extracellular contrast agents or gadoxetic acid-enhanced agents), or imaging assessment of treatment response across locoregional and systemic therapies; (2) Studies evaluating or discussing MRI performed with extracellular contrast agents (ECA-MRI) and/or gadoxetic acid-enhanced MRI, including their roles in lesion characterization, guideline-based diagnosis, or post-treatment assessment; (3) Original research articles, high-quality reviews, international or regional clinical practice guidelines, and expert consensus statements providing clinically relevant insights into diagnostic criteria, imaging biomarkers, or treatment response evaluation; (4) Studies reporting imaging features, diagnostic concepts, or clinical implications relevant to HCC management, irrespective of study design; (5) Publications available in full text and written in English.
Selection process	Shuwei Zhou performed the literature search and initial screening. All authors critically reviewed the selected literature and revised the manuscript based on their respective expertise in abdominal imaging, hepatology, and oncologic treatment of HCC.

## Advantages and limitations of gadoxetic acid-enhanced MRI

**Fig. 1:**
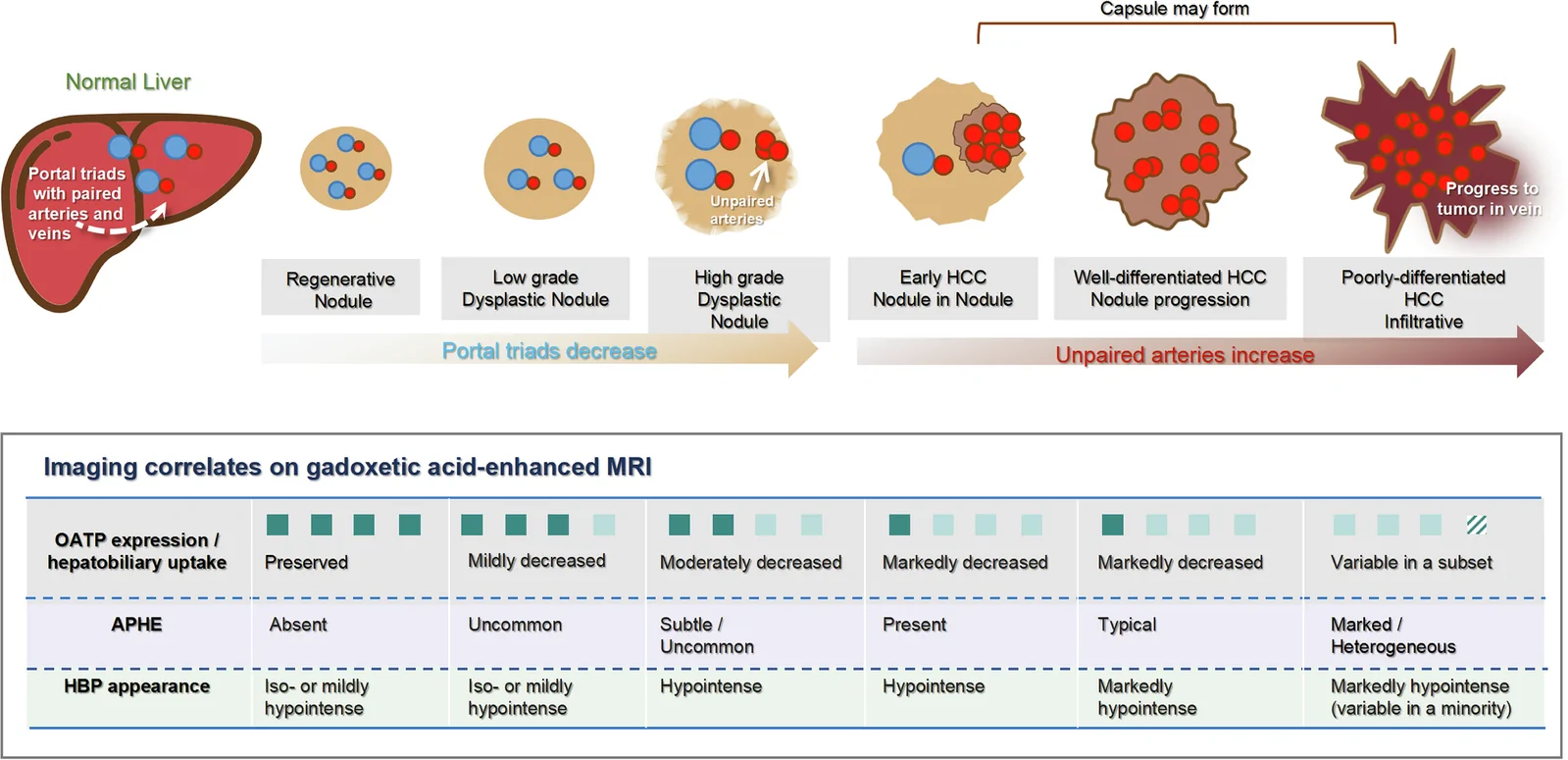
Hepatocarcinogenesis and appearance of imaging features on arterial phase and HBP of gadoxetic acid-enhanced MRI. During hepatocarcinogenesis, downregulation of OATP transporters typically precedes complete neoarterialization, resulting in HBP hypointensity before the development of APHE. Most HCC appear hypointense on HBP, although a small proportion (~5%) with increased OATP expression may appear hyperintense. HCC, hepatocellular carcinoma; OATP, organic anion-transporting polypeptides

Gadoxetic acid-enhanced MRI offers distinct advantages in detecting early-stage HCC, largely because of the temporal sequence of molecular and vascular changes during hepatocarcinogenesis. While APHE, a hallmark of HCC, typically emerges at more advanced stages, downregulation of organic anion-transporting polypeptides (OATP), which mediates gadoxetate uptake, occurs much earlier, even in low-grade dysplastic nodules [3, 13]. As a result, reduced HBP uptake represents an early event, enabling gadoxetic acid-enhanced MRI to identify lesions invisible on conventional dynamic imaging (Fig. 1). Indeed, up to 38% of early HCCs have been reported as detectable exclusively on HBP images [12, 14–16]. Robust parenchymal enhancement during HBP also provides excellent lesion-to-liver contrast, improving detection and accurate delineation.

**Fig. 2:**
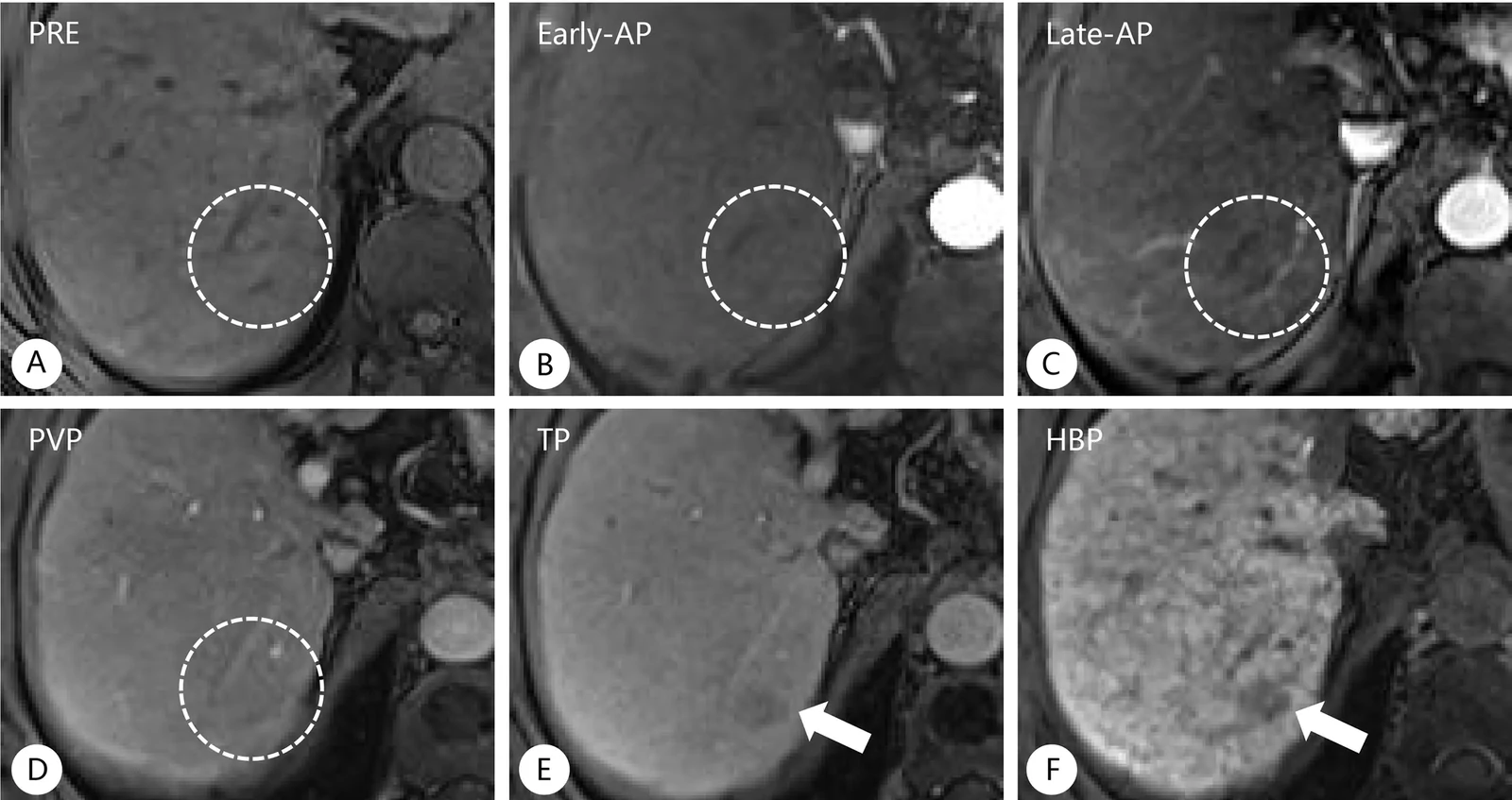
Early detection of a high-grade dysplastic nodule or early HCC on gadoxetic acid-enhanced MRI in a 58-year-old man with a history of HCC. An approximately 1.7-cm lesion in liver segment VII (dashed circles, arrows) was faintly perceptible on the transitional phase image (**E**) as an ill-defined nodule, and became clearly visualized on the HBP image (**F**). The lesion was inconspicuous on T1-weighted precontrast (**A**), arterial phase (**B**, **C**), or portal venous phase (**D**) images. The lesion was initially classified as LR-3 based on the absence of major features but the presence of HBP hypointensity as an ancillary feature favoring malignancy. At 6-month follow-up imaging, the lesion demonstrated significant interval growth, meeting LI-RADS threshold growth criteria and warranting upgrade to LR-4. HCC, hepatocellular carcinoma; TP, transitional phase; HBP, hepatobiliary phase; PRE, precontrast; AP, arterial phase; PVP, portal venous phase; LI-RADS, Liver Imaging Reporting And Data System

HBP hypointensity is increasingly recognized as an independent imaging biomarker of early HCC [17–21]. For small HCCs (10–19 mm), it demonstrates superior sensitivity (84%, *p* < 0.001) compared with major features in the Liver Imaging Reporting and Data System (LI-RADS v2018), including washout appearance, enhancing capsule, and threshold growth, albeit with somewhat lower specificity [19, 22]. Multiple studies consistently confirm that incorporating HBP hypointensity as an adjunct or alternative to washout significantly enhances sensitivity for small HCC detection, with only modest specificity trade-offs [2, 23–37]. However, HBP hypointensity is not pathognomonic for HCC. It can also occur in dysplastic nodules, hemangiomas, and non-hepatocellular malignancies, potentially leading to false-positive diagnoses [38, 39] (Fig. 2).

Despite these diagnostic advantages, gadoxetic acid-enhanced MRI has several limitations relative to extracellular contrast agent-enhanced MRI (ECA-MRI). During the transitional phase (TP) in gadoxetic acid-enhanced MRI, overlap between extracellular and hepatocellular enhancement may blur interpretation, as hypointensity can reflect either true washout appearance or background parenchymal enhancement [40]. This pronounced parenchymal uptake also obscures enhancing capsule visualization, a key LI-RADS v2018 feature, with reported capsule detection rates significantly lower on gadoxetic acid-enhanced MRI than on ECA-MRI (31%–47.4% vs 73%–87.4%) [41–43]. Additionally, the lower gadoxetic acid dose (0.025 mmol/kg vs 0.1 mmol/kg for ECA) and the frequent occurrence of transient severe motion artifacts due to impaired breath-holding may compromise late arterial phase image quality, thereby limiting reliable visualization and assessment of APHE [44, 45]. Multi-arterial phase imaging may partially mitigate this limitation by improving the depiction of APHE on gadoxetic acid-enhanced MRI, enabling APHE detection in 91.4% of HCCs compared with 76.8% with single-arterial phase acquisition, thereby improving the diagnostic performance for HCC [2]. Another important limitation of gadoxetic acid-enhanced MRI is that washout assessment is generally restricted to the portal venous phase (PVP). Because gadoxetic acid-enhanced MRI lacks a true delayed extracellular or equilibrium phase, TP/HBP hypointensity cannot be considered unequivocal washout, which may reduce sensitivity for HCC diagnosis, especially in small lesions without definite PVP washout [34]. Additionally, impaired liver function may also reduce hepatocyte uptake and background liver enhancement on HBP images, decreasing lesion conspicuity [46]. These limitations may hinder identification of LI-RADS major features and potentially reduce diagnostic specificity for definitive HCC diagnosis. Further studies are warranted to clarify how such technical constraints influence diagnostic performance across different guideline frameworks, given the heterogeneous adoption of HBP imaging among international recommendations.

## Gadoxetic acid-enhanced MRI in current practice guidelines

The need to balance the increased sensitivity of HBP imaging against its technical limitations has led to heterogeneous recommendations for gadoxetic acid-enhanced MRI across international guidelines on diagnostics and management of HCC.

Gadoxetic acid-enhanced MRI is widely endorsed as a first-line diagnostic modality for HCC. However, guidelines diverge in their treatment of HBP hypointensity. Asian guidelines, including the Korean Liver Cancer Association–National Cancer Center (KLCA–NCC, v2022, largely consistent with v2018 in definite HCC criteria) [4], the Japan Society of Hepatology (JSH, v2021) [5], and the Asian Pacific Association for the Study of the Liver (APASL, v2017) [6], incorporate HBP hypointensity as a major diagnostic feature, equivalent to washout appearance. In contrast, Western guidelines, such as those from the American Association for the Study of Liver Diseases (AASLD, aligned with LI-RADS v2018) [7], the European Association for the Study of the Liver (EASL, v2018 [47] and v2024 [8]), and the Organ Procurement and Transplantation Network/United Network for Organ Sharing (OPTN/UNOS v2025) [9], consider it only an ancillary feature. Notably, the latest EASL 2024 guidelines recommend adopting LI-RADS v2018 as the diagnostic framework (Table 2 and Supplementary Fig. 1).

**Table 2:** International guidelines for HCC diagnosis using gadoxetic acid-enhanced MRI: comparison between Western and Asian societies

Guideline (year)	High-risk group	Minimum size	Criteria	Definition of washout	Role of HBP hypointensity
AASLD/LI-RADS (v2018)/EASL (v2024)	Cirrhosis OR chronic hepatitis B viral infection OR Current or prior HCC	≥ 10 mm	Nonrim APHE AND: • 10–19 mm with nonperipheral “washout” and no other major features OR • 10–19 mm with ≥ 50% size increase in ≤ 6 months and no other major features OR • ≥ 20 mm with ≥ 1 additional major feature	Hypointensity in PVP/DP on ECA-MRI; Hypointensity in PVP for gadoxetic acid-enhanced MRI	Ancillary feature
EASL (v2018)	PAGE-B^†^: ≥ 18 score point	≥ 10 mm	APHE plus washout	Hypointensity in PVP	Ancillary feature
OPTN/UNOS (v2025)	/	≥ 10 mm	**5A:** 1. Maximum diameter of at least 1 cm and less than 2 cm, as measured on late arterial or portal phase images 2. Non-rim arterial phase hyperenhancement 3. Either of the following: Non-peripheral washout or Biopsy **5A-g:** 1. Maximum diameter of at least 1 cm and less than 2 cm, as measured on late arterial or portal phase images 2. Non-rim arterial phase hyperenhancement 3. Threshold growth defined as size increase of a mass by ≥ 50% in ≤ 180 days on MRI or CT **5B:** 1. Maximum diameter of at least 2 cm and less than or equal to 5 cm, as measured on late arterial or portal phase images 2. Non-rim arterial phase hyperenhancement. 3. One of the following: a. Nonperipheral washout b. Enhancing capsule c. Threshold growth defined as size increase of a mass by ≥ 50% in ≤ 180 days on MRI or CT d. Biopsy	Hypointensity in PVP/DP for ECA-MRI; Hypointensity in PVP for gadoxetic acid-enhanced MRI	Ancillary feature
KLCA–NCC (v2018/2022)	Cirrhosis OR Chronic hepatitis B/C	≥ 10 mm	APHE plus Washout (excluding marked T2 hyperintense OR targetoid lesion)	Hypointensity in PVP/DP/TP/HBP	Major feature (equivalent to washout)
JSH (v2021)	Cirrhosis OR Chronic hepatitis B/C OR Prior HCC	/	APHE plus Washout	Hypointensity in PVP/DP/TP/HBP	Major feature (equivalent to washout)
APASL(v2017)	Cirrhosis OR Chronic hepatitis B/C	/	APHE plus, Washout OR HBP hypointensity (excluding hemangioma)	Hypointensity in PVP/DP/TP	Major feature

*AASLD* American Association for the Study of Liver Diseases, *LI-RADS* liver imaging reporting and data system, *EASL* European Association for the Study of the Liver, *KLCA–NCC* Korean Liver Cancer Association National Cancer Center, *JSH* Japan Society of Hepatology, *APASL* Asian Pacific Association for the Study of the Liver, *OPTN* organ procurement and transplantation network, *HCC* hepatocellular carcinoma, *APHE* arterial phase hyperenhancement, *PVP* portal venous phase, *DP* delayed phase, *TP* transitional phase, *HBP* hepatobiliary phase, *ECA-MRI* extracellular contrast agent-enhanced magnetic resonance imaging, *gadoxetic acid-enhanced MRI* gadoxetic acid-enhanced magnetic resonance imaging

^†^ PAGE-B, platelet, age, gender-hepatitis B score: a risk stratification tool for HCC development in chronic hepatitis B patients, with scores ≥ 18 indicating high risk

These divergent recommendations largely reflect differences in regional epidemiology and clinical priorities. Asian guidelines prioritize sensitivity in HCC detection due to higher disease prevalence, a greater emphasis on early diagnosis and locoregional therapies, and prevalent nationwide surveillance programs targeting high-risk populations. In these contexts, the acceptance of HBP hypointensity as a major imaging feature reflects a deliberate trade-off, accepting modest specificity loss to capture small or atypical lesions earlier [25, 48, 49]. Conversely, Western guidelines prioritize specificity, particularly in contexts such as liver transplantation eligibility and therapeutic decision-making, where false-positive diagnoses may lead to inappropriate interventions or resource misallocation. This conservative stance is reinforced by the recognition that benign or premalignant lesions in cirrhotic livers can also exhibit reduced hepatobiliary uptake, potentially mimicking HCC [39, 50].

Viewed through the lens of the diagnostic workflow, however, these differences extend beyond regional practice patterns and reflect distinct diagnostic intents embedded within each guideline framework. In some settings, imaging is expected to function as an early gatekeeper, identifying lesions that warrant prompt treatment or intensified surveillance. In others, imaging serves as a definitive adjudicator, where diagnostic confirmation directly triggers therapeutic decisions. From this perspective, HBP hypointensity is not inherently a contentious imaging feature; rather, its diagnostic weight is context‑dependent, varying according to the downstream clinical consequences of an HCC diagnosis.

Emerging evidence has prompted ongoing debate about reevaluating the role of HBP hypointensity in Western settings. Multiple studies have demonstrated that upgrading TP or HBP hypointensity to a major diagnostic feature yields substantial gains in sensitivity (approximately 10%–23%) with only modest reductions in specificity (approximately 3%–5%) [19, 26, 27, 35, 40, 51]. Movement toward harmonization is reflected in EASL’s 2024 adoption of LI-RADS, and future guideline iterations may further reconsider the diagnostic status of HBP hypointensity as additional validation data from Western cohorts become available [52, 53].

In the following sections, we examine the diagnostic performance of gadoxetic acid-enhanced MRI under different guideline frameworks, with particular attention to how lesion size–stratified accuracy aligns with these distinct diagnostic intents.

## Diagnostic performance of gadoxetic acid-enhanced MRI of HCC

### Sub-centimeter HCC (< 1 cm): diagnostic uncertainty and surveillance-oriented management

**Fig. 3:**
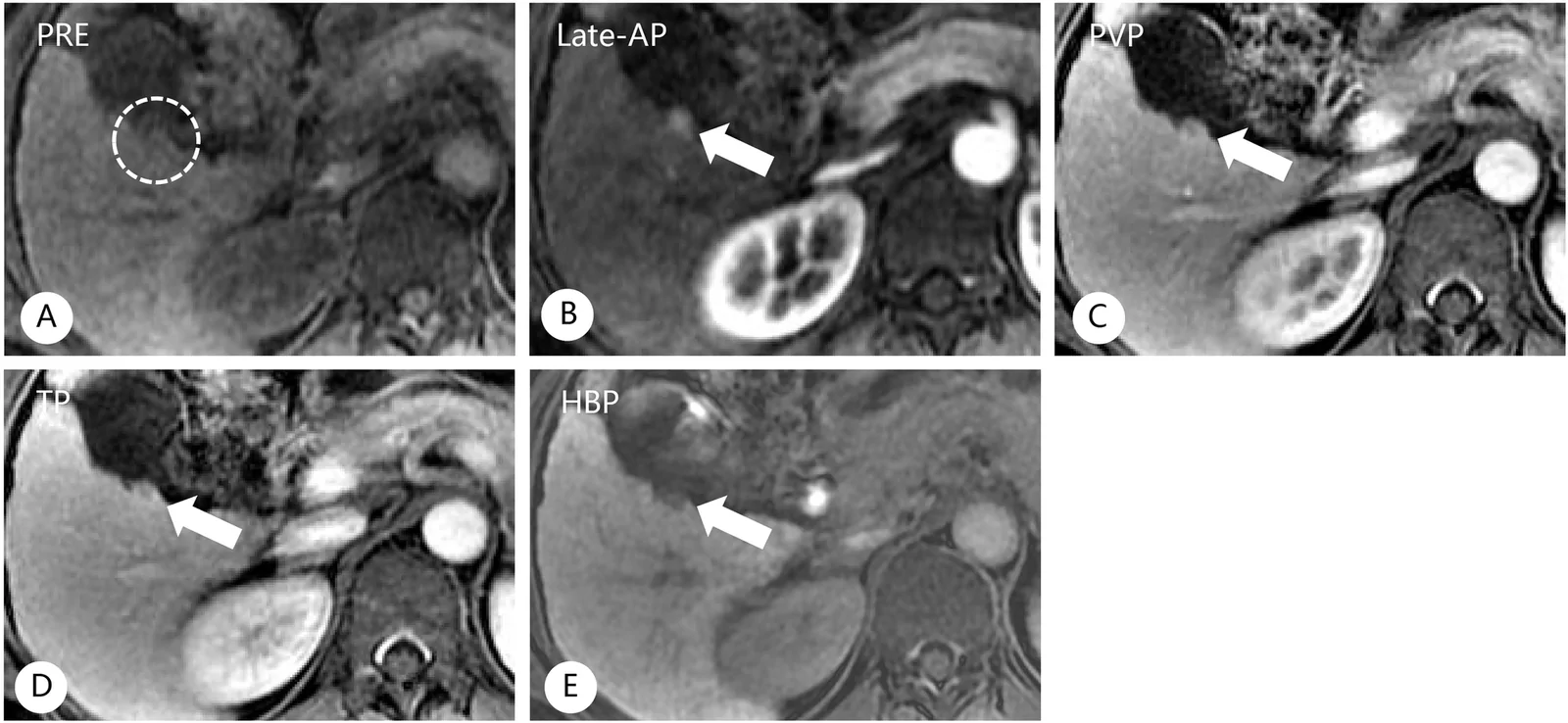
Sub-centimeter HCC on gadoxetic acid-enhanced MRI in a 44-year-old woman with chronic hepatitis B. An approximately 0.8-cm lesion in liver segment VI (arrows, dashed circle in **A**) showed typical imaging features of HCC, with vague hypointensity on precontrast T1-weighted image (**A**), hyperenhancement on arterial phase (**B**), and exhibited washout appearance on the PVP (**C**). The lesion also appeared hypointense on both TP (**D**) and HBP images (**E**). The lesion exhibits major imaging features of HCC but falls below the size threshold for definitive HCC diagnosis according to AASLD/LI-RADS v2018, EASL v2018/2024, OPTN v2025 guidelines, and KLCA–NCC v2018/2022. Conversely, JSH v2021 and APASL v2017 guidelines permit HCC diagnosis regardless of size, classifying this lesion as definitive HCC. Histopathological examination following resection confirmed HCC. AASLD, American Association for the Study of Liver Diseases; LI-RADS, liver imaging reporting and data system; EASL, European Association for the Study of the Liver; OPTN, organ procurement and transplantation network; KLCA–NCC, Korean Liver Cancer Association National Cancer Center; JSH, Japan Society of Hepatology; APASL, Asian Pacific Association for the Study of the Liver; HCC, hepatocellular carcinoma; PRE, precontrast; AP, arterial phase; PVP, portal venous phase; TP, transitional phase; HBP, hepatobiliary phase

The diagnosis of sub-centimeter HCC remains a significant clinical challenge, with varying recommendations across international guidelines on diagnostics and management of HCC. The AASLD/LI-RADS v2018, EASL v2024, and KLCA–NCC v2018/2022 consistently recommend against immediate imaging-based diagnosis for sub-centimeter lesions, advocating instead for short-interval surveillance. This conservative recommendation is supported by evidence demonstrating lower diagnostic sensitivity for sub-centimeter HCCs compared to lesions ≥ 1 cm [37, 54–60], and that the majority of nodules < 1 cm are unlikely to be HCC [61–63]. Conversely, certain guidelines, including the JSH v2021 and APASL v2017, continue to endorse imaging-based diagnosis for typical HCCs without size restrictions (Fig. 3).

Across imaging modalities, diagnostic performance decreases substantially for sub-centimeter HCC [31, 64, 65]. Gadoxetic acid-enhanced MRI shows higher sensitivity than CE-CT and ECA-MRI, with meta-analyses reporting higher sensitivity than CE-CT (46% vs 20%, *p* = 0.03) [60] and ECA-MRI (92% vs 67%, *p* < 0.001). Head-to-head comparative studies further support the advantage of gadoxetic acid-enhanced MRI over CE-CT in this setting [64–66]. However, heterogeneity in diagnostic criteria, mainly centered on APHE and washout appearance, limits direct comparison across studies. Intra-individual comparisons between ECA-MRI and gadoxetic acid-enhanced MRI remain limited and require further investigation.

#### Added value of TP/HBP hypointensity

The diagnostic performance of gadoxetic acid-enhanced MRI for sub-centimeter HCC varies according to guideline criteria. A recent comparative study showed that JSH v2021 achieved higher sensitivity than LI-RADS v2018 (72.0% vs 56.8%, *p* = 0.002) but lower specificity (79.6% vs 92.6%, *p* = 0.001) [21]. This higher sensitivity likely reflects the inclusion of TP/HBP hypointensity as major diagnostic features in JSH v2021, whereas LI-RADS v2018 does not allow definitive HCC diagnosis for sub-centimeter observations.

TP/HBP hypointensity, along with ancillary features such as moderate T2-weighted imaging (T2WI) hyperintensity and restricted diffusion, has been widely investigated to enhance diagnostic accuracy for sub-centimeter HCC [11, 21, 31, 33, 67–74]. TP/HBP hypointensity has emerged as a significant predictor for sub-centimeter HCC diagnosis [21]. A modified diagnostic algorithm incorporating non-rim APHE with washout appearance on PVP or hypointensity on TP demonstrated superior sensitivity (68.2% vs 56.8%, *p* = 0.011) while maintaining comparable specificity (91.7% vs 92.6%, *p* > 0.999) compared to standard LR-4 category [21]. Furthermore, incorporating HBP hypointensity achieved significantly higher sensitivity (78.10% vs 49.64%, *p* < 0.001; 78.10% vs 70.80%, *p* < 0.05) and accuracy (75.32% vs 55.70%, *p* < 0.001; 75.32% vs 71.52%, *p* < 0.05) compared to washout appearance confined to PVP or with the addition of TP hypointensity, respectively. However, this approach resulted in lower specificity (57.14% vs 95.24%, *p* < 0.05) compared to PVP-confined washout appearance assessment [72]. Longitudinal studies have shown that a significant proportion of sub-centimeter lesions identified on TP/HBP demonstrate either histological confirmation of HCC or radiological progression to overt HCC within short follow-up periods [67, 75, 76] (Table 3 and Supplementary Table 1).

**Table 3:** Guideline-stratified diagnostic sensitivity of gadoxetic acid-enhanced MRI for small HCC lesions (≤ 30 mm): comparison with CE-CT/ECA-MRI

Studies	LI-RADS (v2018)		EASL (v2018)	KLCA–NCC (v2018/2022)	APASL (v2017)
	Without HBP/TP hypointensity t	With HBP/TP hypointensity			
Gadoxetic acid-enhanced MRI					
Lee et al [136] (10 mm ≤ size ≤ 19 mm; *n* = 173)	76 [60.5, 87.1]				
Kim et al 37] (TP) (0 mm < size ≤ 30 mm; *n* = 203)	75.3 [68.2, 81.2]	90.9^*^ [85.8, 94.2]			
Kim et al [37] (0 mm < size ≤ 30 mm; *n* = 203)	75.3 [68.2, 81.2]	95.2^*^ [91.2, 97.4]			
Jiang et al [137] (0 mm < size ≤ 20 mm; *n* = 34)	76 [53, 92]		86 [64, 97]		
Hwang et al [138] (TP)^#^ (0 mm < size < 20 mm; *n* = N/A)	43.0^*^ [33.5, 52.5]	64.0^*^ [54.8, 73.2]			
Gaetano et al [139] (0 mm < size ≤ 20 mm; *n* = 43)	88.2 [63.6-98.5]				
Byun et al [96] (10 mm ≤ size ≤ 30 mm; *n* = 493)	71.1 [65.5, 76.1]		71.8 [66.2, 76.8]	85.4 [80.7, 89.0]	86.8 [82.3, 90.3]
Kang et al [20] (10 mm ≤ size ≤ 30 mm; *n* = 385)	56.2 [50.1–62.1]				
Lee et al [140] (10 mm ≤ size ≤ 19 mm; *n* = 104)	51.6 [39.2, 64.1]			75.8 [65.2, 86.5]	
Lee et al [108] (10 mm ≤ size ≤ 19 mm; *n* = 85)	48.0 [34.2, 61.9]		48.0 [34.2, 61.9]		
Ko et al [141] (10 mm ≤ size ≤ 19 mm; *n* = 90)	52.5 [40.0, 64.7]				
Park et al [142] (0 mm < size ≤ 30 mm; *n* = 306)	70.4 [64.6–75.6]				
Jiang et al [143] (0 mm < size ≤ 19 mm; *n* = 200)	61.4				
Lee et al [144] (10 mm ≤ size ≤ 19 mm; *n* = 173)	88.9 [76.0–96.3]			75.6 [60.5–87.1]	
Hwang et al [97]^#^ (10 mm ≤ size ≤ 19 mm; *n* = N/A)	50.1 [39.7, 60.5]		51.0 [40.6, 61.4]	88.8 [81.8, 95.8]	90.9 [84.1, 97.7]
Park et al [94]^#^ (10 mm ≤ size ≤ 30 mm; *n* = 447)	71.1 [68.1, 74.1]		67.9 [64.8, 71.0]	84.9 [82.5,87.3]	89.9 [87.9, 91.9]
Park et al [94]^#^ (10 mm ≤ size ≤ 19 mm; *n* = 249)	64.8 [60.0, 69.6]		63.2 [58.3, 68.1]	83.8 [80.1, 87.5]	85.5 [81.8, 89.2]
Park et al [94]^#^ (20 mm ≤ size ≤ 30 mm; *n* = 198)	75.7 [72.1, 79.3]		71.4 [67.6, 75.2]	85.8 [82.8, 88.8]	91.8 [89.4, 94.2]
Clarke et al [145] (10 mm ≤ size ≤ 19 mm; *n* = 74)	47		46		68
Zhang et al [36] (10 mm ≤ size ≤ 19 mm; *n* = 65)	64.1^*^	79.5^*^			
Xie et al [33] (TP) (10 mm ≤ size ≤ 19 mm; *n* = 174)	55.1^*^ [44.7–65.1]	61.2^*^ [50.8–70.7]			
Huang et al [21]^†^ (0 mm < size < 10 mm; *n* = 240)	56.8 [47.9–65.3]				72.0 [63.4–79.3]
Yoon et al [93] (10 mm ≤ size ≤ 19 mm; *n* = 766)	60.0 [56.2, 63.8]		60.8 [57.0, 64.5]	78.6 [75.2, 81.6]	85.0 [81.8, 87.7]
Lin et al [35] (0 mm < size < 30 mm; *n* = 275)	82.7^*^ [77.1, 87.4]	86.2^*^ [81.0, 90.4]			
Gadoxetic acid-enhanced MRI vs CE-CT					
Min et al [41] (0 mm < size ≤ 19 mm; *n* = 48)	Higher 61.6 [47.9, 73.7]				
Yoon et al [30] (10 mm ≤ size ≤ 19 mm; *n* = 532)	Lower^*^ 62.2 [57.8, 66.4]			Higher^*^ 80.4 [76.7, 83.6]	Higher^*^ 86.8 [83.2, 89.6]
Gadoxetic acid-enhanced MRI vs ECA-MRI					
Kim et al [34] (TP) (10 mm ≤ size ≤ 20 mm; *n* = 48)	Higher^*^ 70.8	Higher* 89.6			
Paisant et al [2] (10 mm ≤ size ≤ 30 mm; *n* = 225)			Lower^*^ 46.4 [38.3, 54.6]	Higher 75.2 [67.5, 81.8]	
Min et al [41] (size ≤ 19 mm; *n* = 84)	Lower 61.6 [47.9, 73.7]				
Min et al [43] (20 mm ≤ size ≤ 30 mm; *n* = 101)	Lower^*^ 71.6 [61.9, 79.6]		Lower^*^ 63.4 [53.6, 72.3]		
Min et al [43] (10 mm ≤ size ≤ 19 mm; *n* = 74)	Lower 62.6 [50.9, 72.9]		Lower 62.6 [50.9, 72.9]		
Yoon et al [92] (10 mm ≤ size ≤ 19 mm; *n* = 53)				Higher^*^ 72.7 [53.5, 83.4]	

*N* number of lesions, *gadoxetic acid-enhanced MRI* gadoxetic acid-enhanced magnetic resonance imaging, *ECA-MRI* extracellular contrast agent-enhanced magnetic resonance imaging, *CE-CT* contrast-enhanced computed tomography, *TP* transitional phase, *HBP* hepatobiliary phase, *LI-RADS* liver imaging reporting and data system, *EASL* European Association for the Study of the Liver, *KLCA–NCC* Korean Liver Cancer Association National Cancer Center, *APASL* Asian Pacific Association for the Study of the Liver

“(TP)” indicates washout assessed on the TP; otherwise, on the HBP

^#^ Results from multiple readers were pooled using a fixed-effect model. When calculation of a weighted average was not feasible, the most representative result was reported.

^*^ Statistically significant (*p* < 0.05)

^†^ For lesions < 10 mm, LI-RADS categorizes them as LR-4

#### Clinical management considerations

The management of sub-centimeter HCCs remains controversial, primarily due to uncertain survival benefits. Analysis of SEER data (2004–2015) and a retrospective cohort study found no significant differences in overall or recurrence-free survival between treated sub-centimeter and 1–2 cm HCCs [61, 77]. Although sub-centimeter lesions exhibiting diagnostic hallmarks on gadoxetic acid-enhanced MRI (APHE, washout, restricted diffusion, T2WI hyperintensity, HBP hypointensity) show lower microvascular invasion and recurrence rates post-resection vs 1–2 cm lesions [78]. Current guidelines recommend short-interval surveillance rather than immediate intervention [79, 80]. Treatment may be reserved for lesions with definitive HCC hallmarks or histological confirmation. Therefore, the diagnosis of sub-centimeter HCC requires a balanced approach, carefully weighing potential clinical benefits against increased healthcare costs and the risk of false-positive diagnoses that may lead to unnecessary interventions.

### Small HCC (≥ 1 cm and < 3 cm): guideline-dependent performance and the role of HBP hypointensity

**Fig. 4:**
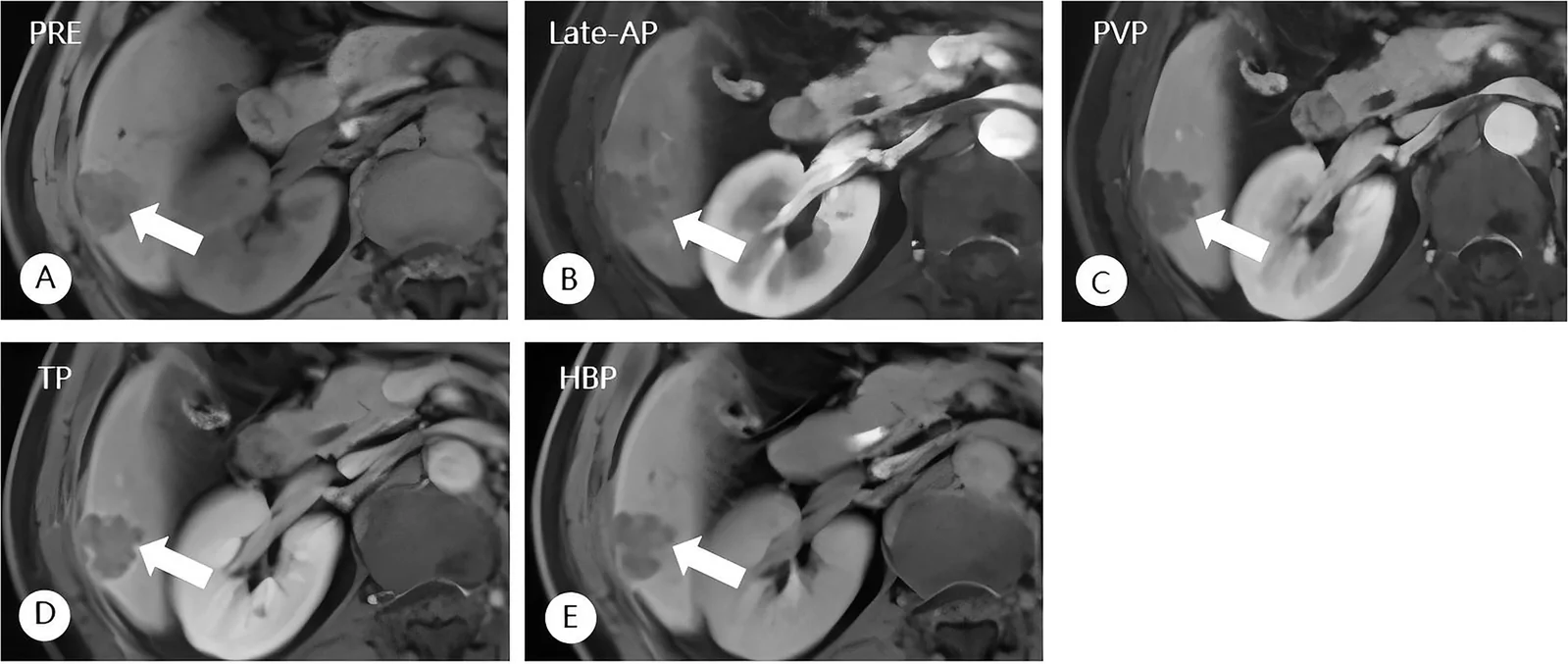
Atypical small HCC (≥ 1 cm and < 3 cm) on gadoxetic acid-enhanced MRI in a 55-year-old man with chronic hepatitis B. A 2.3-cm nodule located at liver segment VI (arrows) demonstrated hypointensity on precontrast T1-weighted imaging (**A**), rim-like APHE (**B**), and hypoenhancement on the PVP (**C**). The lesion remained hypointense on both the transitional (**D**) and HBP (**E**). Due to the absence of non-rim APHE and washout appearance, the lesion did not meet the criteria for definite HCC in any current diagnostic guideline. Histopathological examination following resection confirmed HCC. HCC, hepatocellular carcinoma; PRE, precontrast; AP, arterial phase; PVP, portal venous phase; TP, transitional phase; HBP, hepatobiliary phase

Early detection and treatment of small HCC ≥ 1 cm represents a critical point in clinical management, as patients with small HCC (early stage) demonstrate significantly superior survival benefit from surgical resection or locoregional therapies compared to those with larger tumors [81–84]. However, the imaging diagnosis of small HCCs remains challenging because these lesions frequently exhibit incomplete or atypical vascular hallmarks, resulting in variable diagnostic performance across imaging modalities and guideline frameworks [85–88] (Fig. 4).

#### Guideline-dependent performance of gadoxetic acid-enhanced MRI

Gadoxetic acid-enhanced MRI generally shows higher sensitivity than CE-CT for detecting small HCC, with comparable specificity in many meta-analyses and comparative studies [20, 30, 55, 60, 89–91]. However, these pooled results should be interpreted cautiously because diagnostic criteria are not always stratified. When the same modality is assessed under different guideline frameworks, performance can change substantially.

Recent guideline-specific comparative studies have revealed this critical knowledge gap. When evaluated using LI-RADS v2018 criteria, head-to-head comparative studies have yielded conflicting results. Min et al demonstrated that gadoxetic acid-enhanced MRI achieved higher sensitivity than CE-CT for diagnosing small HCCs (≤ 19 mm), though this difference did not reach statistical significance (84 lesions analyzed) [41]. Conversely, another large-scale study of 532 lesions (10–19 mm) found that gadoxetic acid-enhanced MRI demonstrated lower sensitivity than CE-CT when applying identical LI-RADS v2018 criteria [30]. Strikingly, the same imaging modalities exhibited reversed diagnostic performance when evaluated under different guideline frameworks. Using APASL v2017 and KLCA–NCC v2018/2022 criteria, gadoxetic acid-enhanced MRI achieved significantly superior sensitivity (80.4%–86.8%) and accuracy (82.0%–82.8%) compared to CE-CT [30]. This dramatic performance reversal underscores the fundamental role of TP/HBP hypointensity, which is incorporated as a major feature in Asian-Pacific guidelines but not in LI-RADS.

Similar guideline-dependent patterns were observed when comparing gadoxetic acid-enhanced MRI with ECA-MRI. While meta-analyses consistently favor gadoxetic acid-enhanced MRI, particularly for small HCCs [9, 26, 78], guideline-stratified head-to-head comparative studies reveal a more nuanced picture that challenges these aggregated findings. Using guidelines that exclude HBP hypointensity, gadoxetic acid-enhanced MRI often demonstrates comparable or inferior performance. When applying LI-RADS v2018 criteria, only one study found significantly higher sensitivity for gadoxetic acid-enhanced MRI compared to ECA-MRI [34], while multiple investigations reported opposite findings [2, 41, 43]. Similarly, EASL v2018-based evaluations showed lower sensitivity for gadoxetic acid-enhanced MRI than ECA-MRI [2, 43]. However, guidelines incorporating HBP hypointensity consistently favor gadoxetic acid-enhanced MRI. When evaluated using KLCA–NCC v2018/2022 or JSH v2014 criteria, gadoxetic acid-enhanced MRI demonstrated significantly higher sensitivity than ECA-MRI [2, 92]. However, specificity was generally comparable under LI-RADS-based criteria and comparable or lower when HBP-inclusive criteria were applied, indicating that the apparent advantage of gadoxetic acid-enhanced MRI reflects a criterion-dependent sensitivity–specificity trade-off rather than uniform diagnostic superiority (Supplementary Table 1).

#### Role of HBP hypointensity and optimized algorithms

International guidelines differ substantially in how they use HBP hypointensity. Western guidelines, including AASLD/LI-RADS v2018, EASL v2024, and OPTN/UNOS v2025, explicitly exclude HBP hypointensity as a major diagnostic feature for HCC diagnosis. In contrast, Asian society guidelines, including KLCA–NCC v2018/2022, JSH v2021, and APASL v2017, incorporate HBP hypointensity as a major feature equivalent to washout appearance [30, 93, 94].

These differences explain much of the variation in reported diagnostic performance. A comprehensive meta-analysis demonstrated that KLCA–NCC v2018/2022 achieved significantly higher sensitivity than LI-RADS v2018 when using gadoxetic acid-enhanced MRI for small HCCs (81% vs 55%), while maintaining comparable specificity [95]. This superiority has been consistently validated across multiple cohort studies, where Asian guidelines (KLCA–NCC v2018/2022: sensitivity 80.4%–88.8%, specificity 78.4%–89.1%; APASL v2017: sensitivity 86.8%–91.8%, specificity 73.2%–86.9%) demonstrated higher sensitivity but lower specificity compared to Western guidelines (LI-RADS v2018: sensitivity 50.1%–75.7%, specificity 83.7%–95.5%; EASL v2018: sensitivity 51.0%–71.8%, specificity 83.7%–96.4%) for small HCC diagnosis [30, 93, 94, 96, 97]. These performance disparities reflect carefully considered adaptations to distinct epidemiological and therapeutic landscapes rather than arbitrary regional preferences. In regions with high HCC prevalence and frequent use of locoregional therapy, earlier detection may be prioritized. In settings where liver transplantation and organ allocation are central, higher specificity is often emphasized [84, 98]. This contextual framework suggests that each guideline represents an optimal diagnostic strategy for its intended clinical environment, with performance metrics appropriately weighted according to regional disease burden, therapeutic options, and healthcare system priorities.

Recent studies have therefore explored optimized gadoxetic acid-enhanced MRI algorithms that combine HBP hypointensity with APHE, diffusion restriction, or other ancillary features [26, 48]. These algorithms may improve sensitivity, especially for lesions ≤ 2 cm, but most remain insufficiently validated. The development of such evidence-based, HBP-inclusive diagnostic frameworks represents a potential pathway toward harmonizing the demonstrated clinical value of this imaging feature with standardized diagnostic protocols for small HCCs.

#### Toward a pragmatic, intent-aligned diagnostic strategy for small HCC

Rather than treating HBP hypointensity as uniformly major or ancillary, evidence from small HCC cohorts supports a more nuanced, context-aware application. In sensitivity-oriented scenarios, such as surveillance-detected nodules in patients eligible for curative local therapies, HBP hypointensity may justifiably serve as a decisive feature to upgrade lesions that lack complete vascular hallmarks. Conversely, in specificity-critical settings, HBP hypointensity is best interpreted as a complementary feature that increases diagnostic confidence without independently establishing a definitive diagnosis. Importantly, this intent-aligned approach does not necessitate formal modification of existing guideline criteria. Instead, it encourages clinicians to interpret gadoxetic acid-enhanced MRI findings within the broader clinical decision pathway, aligning diagnostic stringency with therapeutic consequence. Such a strategy reconciles the demonstrated sensitivity advantages of gadoxetic acid-enhanced MRI for small HCC with the legitimate concerns regarding specificity that underpin Western diagnostic frameworks.

### Across the full HCC spectrum: diminishing incremental value of HBP findings with increasing tumor size

When HCC of all sizes is considered, the diagnostic performance of gadoxetic acid-enhanced MRI demonstrates less variability across international HCC diagnostic and management guidelines than that observed for small lesions. Subsequent analyses incorporating guideline-based subgroup evaluation have revealed more informative patterns.

#### Diagnostic performance according to different criteria

Using LI-RADS v2018 criteria, contrast-enhanced MRI achieved pooled sensitivity and specificity of 73% (95% CI: 65%–80%) and 92% (95% CI: 89%–95%), respectively. Performance with EASL v2018 criteria was comparable (sensitivity: 67%, 95% CI: 51%–80%; specificity: 91%, 95% CI: 84%–95%), while KLCA–NCC v2018/2022 criteria demonstrated the highest sensitivity (78%, 95% CI: 72%–82%) with maintained high specificity (90%, 95% CI: 86%–93%) [99]. These meta-analytic findings have been consistently validated in multiple cohort studies. Western guidelines, including LI-RADS v2018 (sensitivity: 29.1%–70.6%; specificity: 89.6%–100%) and EASL v2018 (sensitivity: 26.2%–69.3%; specificity: 71.4%–100%), consistently exhibited lower sensitivity but higher specificity compared to Asian guidelines such as KLCA–NCC v2018/2022 (sensitivity: 65.5%–85.0%; specificity: 84.1%–92.1%) and APASL v2017 (sensitivity: 75.7%–92.0%; specificity: 52.2%–100%) when using gadoxetic acid-enhanced MRI [30, 93, 97, 100, 101].

Despite the relatively low sensitivity of gadoxetic acid-enhanced MRI demonstrated above, meta-analysis revealed that gadoxetic acid-enhanced MRI exhibited higher sensitivity than CE-CT (85% vs 68%, *p* < 0.05) with comparable specificity. However, head-to-head comparative studies evaluating gadoxetic acid-enhanced MRI vs CE-CT for HCC detection have yielded conflicting results. When using LI-RADS v2018, most studies report superior sensitivity for gadoxetic acid-enhanced MRI compared to CE-CT with comparable or lower specificity [41, 91, 102], although some studies showed contrary results [30, 103]. When using KLCA–NCC v2018/2022 and APASL v2017, gadoxetic acid-enhanced MRI consistently demonstrated higher sensitivity than CE-CT with comparable or lower specificity [30], likely due to the diagnostic value of HBP hypointensity as a major imaging feature as illustrated above (Supplementary Tables 2 and 3, and Supplementary Fig. 1).

When compared with ECA-MRI, meta-analyses demonstrated no difference in diagnostic performance between gadoxetic acid-enhanced MRI (sensitivity: 75.0%–76.0%; specificity: 90%–92%) and ECA-MRI (sensitivity: 70.0%–72.0%; specificity: 92.0%–94%) regardless of diagnostic criteria employed [99, 104]. Similarly, no difference was observed in HCC detection rates across LI-RADS v2018 categories [105]. Nevertheless, cohort studies suggest that gadoxetic acid-enhanced MRI may show lower sensitivity than ECA-MRI under Western criteria such as LI-RADS and EASL, but higher sensitivity under HBP-inclusive KLCA–NCC criteria without compromising specificity [41, 43, 106–108]. These findings indicate that the apparent advantage of gadoxetic acid-enhanced MRI depends not only on contrast agent type, but also on diagnostic criteria, lesion size distribution, and the weighting of HBP findings [92, 109] (Supplementary Tables 2 and 3, and Supplementary Fig. 1).

#### Diagnostic intent and diminishing guideline divergence in advanced disease

Importantly, when HCCs of all sizes are considered, the clinical impact of diagnostic intent-driven divergence among guidelines becomes less pronounced. As tumor size increases, characteristic vascular hallmarks, APHE, washout appearance, and capsule appearance, are more consistently expressed, reducing reliance on ancillary or functional imaging features such as HBP hypointensity. Consequently, diagnostic concordance across guideline frameworks naturally increases in larger or more advanced tumors. From an intent-based perspective, this convergence reflects the fact that both sensitivity-oriented and specificity-oriented diagnostic strategies are more readily satisfied once classical imaging features are present. In such settings, the incremental value of HBP imaging is attenuated, and the performance of gadoxetic acid-enhanced MRI becomes less dependent on how HBP findings are weighted within individual guidelines.

These observations suggest that efforts toward pragmatic harmonization of HCC imaging diagnosis should primarily focus on early-stage and small lesions, where diagnostic uncertainty is greatest and where the intended clinical role of imaging varies most substantially. For HCC of all sizes, particularly advanced disease, gadoxetic acid-enhanced MRI performs robustly across guideline frameworks, and differences in diagnostic intent exert a comparatively minor influence on overall accuracy.

**Fig. 5:**
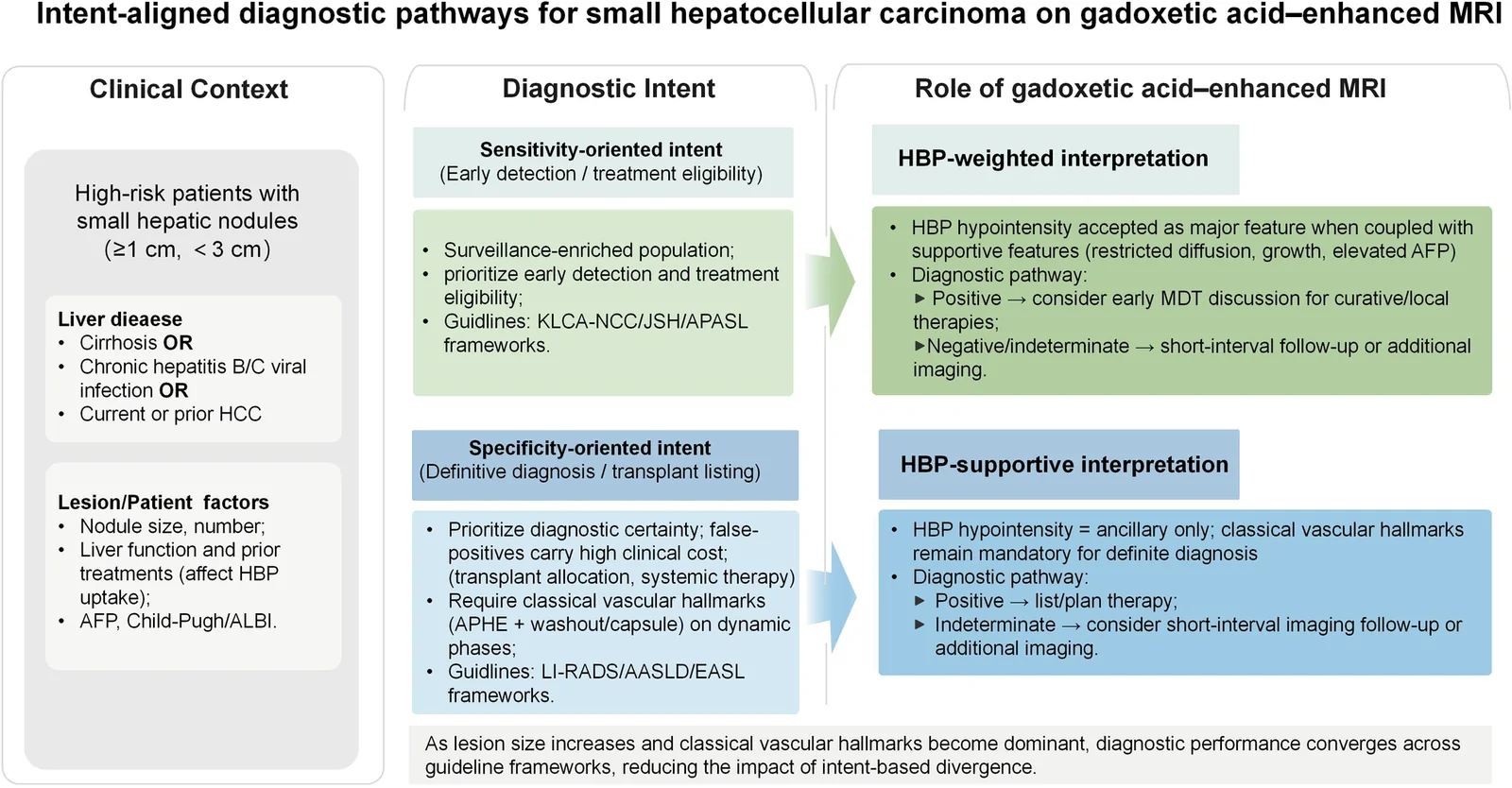
Intent-aligned diagnostic pathways for small HCC on gadoxetic acid-enhanced MRI. HCC, hepatocellular carcinoma; DWI, diffusion-weighted imaging; AFP, α-fetoprotein; HBP, hepatobiliary phase; KLCA–NCC, Korean Liver Cancer Association National Cancer Center; JSH, Japan Society of Hepatology; APASL, Asian Pacific Association for the Study of the Liver; AASLD, American Association for the Study of Liver Diseases; LI-RADS, liver imaging reporting and data system; EASL, European Association for the Study of the Liver

Accordingly, the value of gadoxetic acid-enhanced MRI in this context lies less in resolving guideline discordance and more in providing comprehensive lesion characterization within established diagnostic pathways. This natural convergence underscores that diagnostic intent–aligned interpretation is most critical at the early stages of hepatocarcinogenesis, while advanced disease benefits from greater intrinsic imaging agreement regardless of guideline framework (Fig. 5).

## Gadoxetic acid-enhanced MRI in assessment of treatment response

### Conceptual framework: why treatment response assessment is fundamentally different from primary diagnosis

**Fig. 6:**
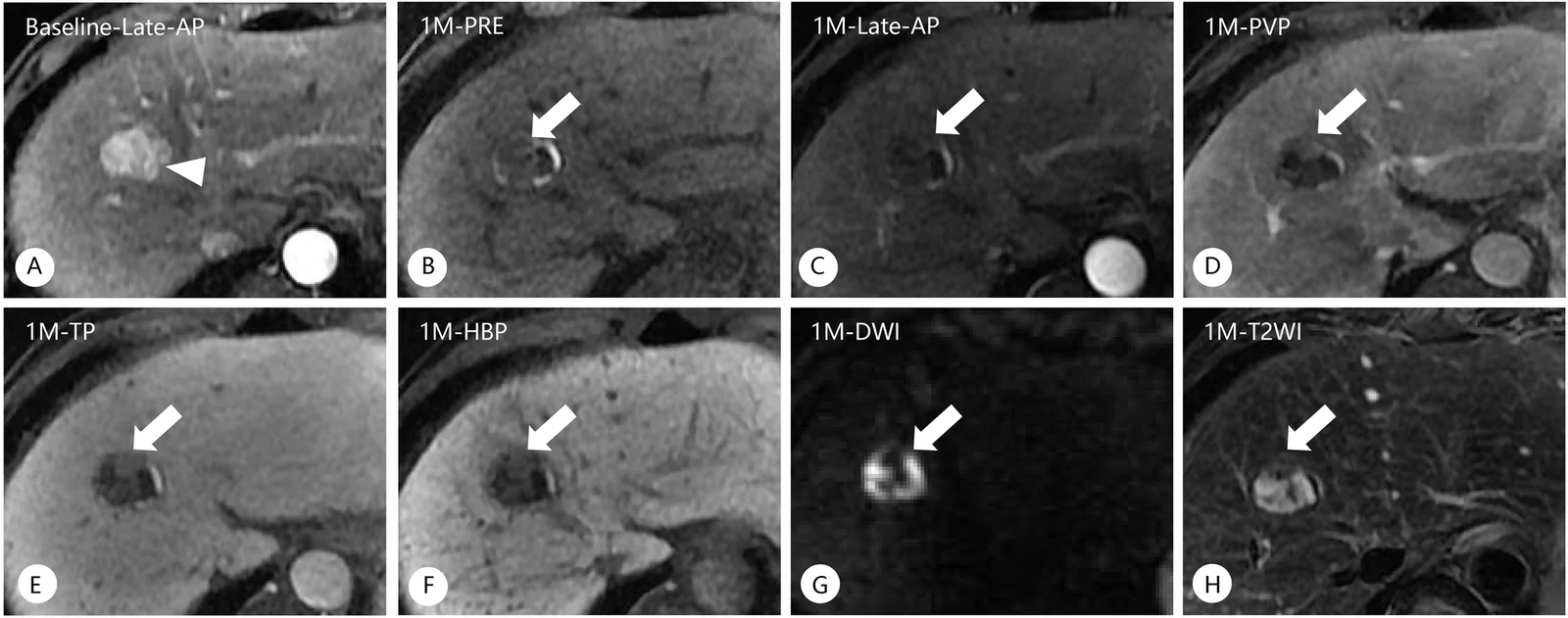
HCC (3.3 cm) in liver segment VIII treated with conventional transarterial chemoembolization in a 58-year-old man with chronic hepatitis B (**A**, arrowhead). One month after treatment, gadoxetic acid-enhanced MRI demonstrated predominantly iso- to hypointensity with peripheral rim hyperintensity on precontrast T1-weighted imaging (**B**). On the late arterial phase (**C**), a 1.4-cm nodular area of mild hypointensity was observed within the treated lesion. The mass-like area showed iso- to hypointensity on the portal venous (**D**), transitional (**E**), and hepatobiliary (**F**) phases, without diffusion restriction (**G**) or T2WI hyperintensity (**H**). The lesion was categorized as mRECIST-nonviable and LR-TRA Equivocal (v2017 and v2024). Although ancillary features, such as mild-to-moderate T2 hyperintensity and diffusion restriction, are considered in LR-TRA v2024, both were negative in this case. Subsequent follow-up confirmed the lesion as nonviable. HCC, hepatocellular carcinoma; PRE, precontrast; AP, arterial phase; PVP, portal venous phase; TP, transitional phase; HBP, hepatobiliary phase; mRECIST, modified response evaluation criteria in solid tumors; LI-RADS TRA, liver imaging reporting and data system treatment response algorithm

Unlike primary diagnosis, treatment response assessment in HCC is not merely a question of lesion detection but of distinguishing viable tumor from treatment-induced parenchymal alterations. Accurate evaluation of treatment responses is essential for guiding retreatment strategies, determining transplant eligibility, and stratifying prognosis in patients with HCC. Current clinical criteria for assessing treatment response in HCC primarily include the Response Evaluation Criteria in Solid Tumors (RECIST) 1.1 [110], the immune-modified RECIST (iRECIST) 111], the modified RECIST (mRECIST), the EASL criteria [112], and LI-RADS Treatment Response Algorithm (LI-RADS TRA) [7, 113, 114] (Tables 4 and 5, Fig. 6, and Supplementary Fig. 1).

**Table 4:** Definitions of tumor response criteria for HCC

Category	EASL	RECIST 1.1	mRECIST	Category	LR-TRA 2018	LR-TRA 2024	
						Nonradiation	Radiation
Complete response (CR)	Disappearance of all viable lesions	Disappearance of all target lesions (up to two measurable liver lesions)	Disappearance of all viable lesions (up to two measurable liver lesions)	Nonviable	• No lesional enhancement OR • Treatment-specific expected enhancement pattern	No mass-like enhancement present	No mass-like enhancement present
Partial response (PR)	≥ 50% ↓ in the sum of the product of bi-dimensional diameters of viable lesions (vs baseline sum of diameters of viable lesions)	≥ 30% ↓ in the sum of the greatest one-dimensional diameters of target lesions (vs baseline sum of the diameters of target lesions)	≥ 30% ↓ in the sum of the greatest one-dimensional diameters of viable lesions (vs baseline sum of the diameters of viable lesions)	Equivocal	Enhancement atypical for treatment-specific expected enhancement pattern and not meeting criteria for probably or definitely viable	Uncertainty about mass-like enhancement (presence or morphology)	
Progressive disease (PD)	≥ 25% ↑ in the sum of the diameters of viable lesions (vs smallest sum of the diameters of viable lesions recorded since treatment started)	≥ 20% ↑ in the sum of the diameters of target lesions (vs smallest sum of the diameters of the target lesions recorded since treatment started)	≥ 20% ↑ in the sum of the diameters of viable lesions (vs smallest sum of the diameters of the viable lesions recorded since treatment started)	Nonprogressing			Mass-like enhancement, which is stable or decreased in size over time after LRT
Stable disease (SD)	Any case that does not qualify for either PR or PD	Any case that does not qualify for either PR or PD	Any case that does not qualify for either PR or PD	Viable	Nodular, masslike, or thick irregular tissue in or along the treated lesion with any of the following: • APHE OR • Washout appearance OR • Enhancement similar to pretreatment	Masslike enhancement (any degree, any phase)	Mass-like enhancement, which is new or increased in size over time after LRT

Viable lesions: enhancing lesion on arterial phase

*LRT* locoregional therapy, ↓ decrease, ↑ increase, *EASL* European Association for the Study of the Liver, *RECIST* response evaluation criteria in solid tumors, *LI-RADS TRA* liver imaging reporting and data system treatment response algorithm

**Table 5:** Diagnostic sensitivity of gadoxetic acid-enhanced MRI for residual or recurrent HCC after locoregional treatment

Studies	Locoregional treatment	Diagnostic criteria	Gadoxetic acid-enhanced MRI (sen.)		ECA (sen.)	CE-CT (sen.)
			Without TP/HBP	With TP/HBP		
Watanabe et al [121]^#^ (*n* = 61)	RFA	• Nodular hypointensity on T1WI AND • Nodular hyperintensity on T2WI AND • Nodular enhancement on AP AND • Washout on PVP With/Without • Hypointensity on HBP	70 [52, 83]	73 [56, 86]		
Schelhorn et al [122] (*n* = /)	Radioembolization	• Arterial enhancement AND • Washout OR • Hypointensity on PVP/DP OR TP/HBP	78.6	92.9	64.3	
Rimola et al [124] (*n* = 19)	TACE	EASL	81 [53, 93]		95 [88, 98]	
Rimola et al [124] (*n* = 19)	RFA	EASL	80 [62, 89]		98 [96, 99]	
Kim et al [123] (*n* = 183)	TACE/RFA	• LI-RADS TRA v2018-viable With/Without Hypointensity on TP	66.7^*^	77.4		
Yim et al [125] (*n* = 136)	TACE/RFA	• Focal, discrete, nodular arterial hyperenhancement AND • Washout on PVP OR hypointensity on TP/HBP		77.7	91.3^*^	
Rimola et al [126] (*n* = 53)	Ablation/Resection	Hypointensity on HBP		69.05		
Park et al [118] (*n* = 138)	TACE/RFA/PEIT	LI-RADS TRA v2018-Viable With/Without Hypointensity on HBP/restricted diffusion/intermediate hyperintensity on T2WI	76^*^ [67, 84]	84 [76, 91]		73^*^ [64, 81]
Park et al [118] (*n* = 138)	TACE/RFA/PEIT	Hypointensity on HBP		81 [72, 88]		
Youn et al [127] *(n* = 105)	TACE/RFA/PEA	LI-RADS TRA v2018-viable	63.0 [50.9, 74.0]			
Youn et al [127] *(n* = 105)	TACE	LI-RADS TRA v2018-viable	67.4 [51.5, 80.9]			
Liu et al [146] (*n* = 74)	DEB-TACE	• Arterial hyperenhancement AND • Hypointensity on PVP/DP OR HBP		96.97		

*N* number of lesions, *TACE* transarterial chemoembolization, *RFA* radiofrequency ablation, *PEIT* percutaneous ethanol injection therapy, *PEA* percutaneous ethanol ablation, *LI-RADS TRA* liver imaging reporting and data system treatment response algorithm, *EASL* European Association for the Study of the Liver, *AP* arterial phase, *PVP* portal venous phase, *DP* delay phase, *TP* transitional phase, *HBP* hepatobiliary phase

^*^ Statistically significant (*p* < 0.05) compared to gadoxetic acid-enhanced MRI using TP/HBP images

^#^ Results from multiple readers were pooled using a fixed-effect model. When calculation of a weighted average was not feasible, the most representative result was reported

While RECIST and iRECIST serve as the regulatory standard for systemic therapy trials with broad applicability, both exhibit inherent limitations in capturing necrosis-predominant responses characteristic of locoregional therapies. To overcome this, enhancement-based criteria have been developed to more accurately quantify viable tumor burden. EASL criteria utilize bidimensional measurements of arterial phase hyperenhancing components. mRECIST further refines this approach employing unidimensional measurements of arterial hyperenhancing viable tumor, while maintaining RECIST 1.1 rules for atypical and extrahepatic disease. Given its robust correlation with survival outcomes, mRECIST has emerged as the preferred radiologic endpoint in clinical trials evaluating locoregional or combination therapies [115, 116]. In contrast to these patient-level assessment systems, the LI-RADS TRA, introduced in 2017 and updated in 2024, represents the first standardized lesion-based framework. LR-TRA v2024 is stratified into radiation therapy [26] and nonradiation cores, categorizing individual lesions as Nonviable, Viable, Equivocal, Nonprogressing, or Nonevaluable based on enhancement patterns and temporal changes. This algorithm was specifically designed for clinical decision-making and standardized reporting [7, 113, 114].

Currently, all these criteria rely primarily on dynamic enhancement features and exclude TP/HBP findings from gadoxetic acid-enhanced MRI. Gadoxetic acid-enhanced MRI introduces an additional, biologically distinct dimension: hepatocellular function, as reflected by TP/HBP signal alterations. This functional information has the potential to complement enhancement-based criteria by capturing early hepatocyte injury, persistent viable tumor foci, and spatial heterogeneity within treated lesions. The clinical relevance of this added dimension, however, is highly treatment-specific, necessitating a stratified evaluation rather than a uniform endorsement of gadoxetic acid-enhanced MRI across all therapeutic settings.

### Nonradiation locoregional treatment response assessment

Contrast-enhanced MRI has demonstrated more reliable assessment of residual viable tumor after conventional TACE compared with CE-CT [10, 117, 118]. However, the role of gadoxetic acid-enhanced MRI in treatment response evaluation remains debated and insufficiently studied [119]. A meta-analysis reported comparable diagnostic performance of gadoxetic acid-enhanced MRI and ECA-MRI for assessing treatment response after locoregional therapy in HCC [120]. However, it neither standardized diagnostic criteria nor specifically evaluated the added value of TP/HBP or the overall diagnostic performance of the full gadoxetic acid-enhanced MRI protocol.

With respect to the added value of TP/HBP imaging, several studies have shown that the sensitivity of gadoxetic acid-enhanced MRI without using TP/HBP hypointensity as an ancillary feature ranges from 63% to 81%, whereas incorporating TP/HBP hypointensity increases sensitivity to 69.05%–96.97% [118, 121–127]. Furthermore, across cohorts in which gadoxetic acid-enhanced MRI was performed, adding TP/HBP hypointensity as an ancillary feature consistently improved the sensitivity of gadoxetic acid-enhanced MRI, regardless of the diagnostic algorithm [121–123]. Importantly, the added value of TP/HBP imaging does not necessarily translate into superior overall performance of gadoxetic acid-enhanced MRI. An intraindividual comparative study demonstrated that ECA-MRI achieved significantly higher diagnostic sensitivity (91.3%) compared to gadoxetic acid-enhanced MRI (77.7%) when the latter employed the diagnostic criteria of “focal, discrete, nodular APHE and washout on PVP or hypointensity on TP/HBP” [125]. ECA-MRI maintained superior sensitivity even when gadoxetic acid-enhanced MRI did not utilize TP/HBP hypointensity as an ancillary feature [125]. The higher sensitivity of ECA‑MRI may be attributable to its superior detection of APHE, whereas gadoxetic acid-enhanced MRI has the disadvantage of the lower administered contrast dose and its greater susceptibility to motion artifacts, both of which can compromise late arterial phase image quality [128, 129]. In addition, repeated locoregional treatments may exacerbate hepatic dysfunction and diminish lesion conspicuity on the HBP by reducing enhancement of the background liver parenchyma. Perfusion alterations induced by locoregional therapies may also appear as HBP hypointensity, potentially resulting in false positive findings on gadoxetic acid-enhanced MRI [130]. Notably, only one earlier study reported lower sensitivity for ECA than for gadoxetic acid-enhanced MRI, with or without consideration of TP/HBP hypointensity; however, these differences did not reach statistical significance. It should also be emphasized that this study used a reference standard of contrast‑enhanced CT combined with either α-fetoprotein or γ-glutamyltransferase, which may be suboptimal.

### Radiation locoregional and systemic treatment response assessment

Emerging treatment modalities, including radiation-based therapies (such as transarterial radioembolization [TARE] and stereotactic body radiation therapy [SBRT]), immune checkpoint inhibitors, molecular targeted agents, and various combination regimens, exhibit distinct response kinetics that challenge traditional imaging assessment paradigms [131].

After radiation-based locoregional therapy, the treated lesion and adjacent liver parenchyma often show a prolonged and complex evolution of enhancement patterns and signal abnormalities, including persistent mass-like APHE and heterogeneous perfusion changes that can closely mimic viable tumor [113]. Gadoxetic acid-enhanced MRI offers several potential advantages. Radiation-induced hepatocyte injury and fibrosis within the treatment volume typically appear as geographic or segmental hypointensity on TP/HBP images, helping to delineate the irradiated zone. In contrast, residual or recurrent HCC more commonly manifests as focal, nodular HBP hypointensity that is spatially concordant with mass-like enhancement and ancillary features such as diffusion restriction or mild–moderate T2WI hyperintensity [132, 133]. LI-RADS Radiation TRA v2024 has been specifically adapted for this setting, introducing the “LR-TR Nonprogressing” category and allowing application of ancillary features favoring viability to upgrade lesions from LR-TR Nonprogressing to LR-TR Viable. However, successfully treated tumors may remain stable in size and enhancement for 3–6 months or longer, and complete necrosis is uncommon after SBRT or TARE performed with standard dosimetry. Consequently, a clear, direct superiority of gadoxetic acid-enhanced MRI over CE-CT or ECA-MRI for post-radiation response assessment has not yet been demonstrated.

Accurate imaging-based response assessment in HCC treated with contemporary systemic therapies, including molecular targeted agents and immune checkpoint inhibitor–based regimens, remains challenging because atypical treatment patterns are not fully captured by conventional size-based criteria such as RECIST 1.1 [8, 134]. Anti-angiogenic tyrosine kinase inhibitors and VEGF-directed combinations frequently induce intratumoral necrosis and reduced arterial phase enhancement without substantial tumor shrinkage. Accordingly, criteria that focus on viable, enhancing tumor, such as mRECIST, tend to better reflect antitumor activity, although they still face limitations in the setting of heterogeneous enhancement, infiltrative disease, and multifocal progression under prolonged systemic therapy. Immunotherapy introduces additional complexity, including delayed responses, and phenomena such as pseudoprogression and hyperprogression, all of which may lead to misclassification of clinical benefit when applying standard RECIST or purely enhancement-based criteria. In a cohort of patients receiving immunotherapy, Zhou et al reported that mRECIST had superior discriminatory ability for differentiating responders from nonresponders compared with RECIST 1.1 and iRECIST [135]. However, standardized imaging response criteria specifically tailored to the unique kinetic and morphologic patterns of contemporary systemic therapies are still lacking, underscoring the need for continued efforts in this area.

TP/HBP signal intensity alterations within and adjacent to treated lesions on gadoxetic acid-enhanced MRI may reveal early necrosis, residual viable foci, and intratumoral heterogeneity, even when overall tumor size or APHE remains largely unchanged. However, current evidence is insufficient to justify routine integration of TP/HBP findings into standardized response assessment criteria. Interpretation may be confounded by treatment-related hepatic dysfunction, immune-mediated hepatitis, and alterations in background parenchymal enhancement, thereby reducing specificity. At present, gadoxetic acid-enhanced MRI should be considered a problem-solving and complementary modality rather than a substitute for established enhancement-based criteria for systemic therapy response evaluation.

## Conclusions

Rather than advocating a single superior imaging modality or diagnostic criterion, this review reframes the role of gadoxetic acid-enhanced MRI within an intent-aligned, strategy‑oriented diagnostic paradigm. By systematically demonstrating how identical imaging findings yield divergent diagnostic performance under different guideline frameworks, it shifts the debate from modality-centered comparison to contextual optimization of diagnostic strategies. The clinical value of gadoxetic acid-enhanced MRI is maximized when interpreted in alignment with diagnostic intent, particularly for small hepatocellular carcinoma, where diagnostic uncertainty is greatest and guideline divergence is most pronounced, whereas diagnostic convergence naturally increases in advanced disease as classical vascular hallmarks become dominant. In this sense, the strength of gadoxetic acid-enhanced MRI lies not in universal superiority, but in its selective, purpose-driven deployment, informed by regional epidemiology, therapeutic pathways, and acceptable trade‑offs between sensitivity and specificity, thereby positioning gadoxetic acid-enhanced MRI as a key tool for precision diagnosis in HCC.

## Supplementary information

ELECTRONIC SUPPLEMENTARY MATERIAL

## Data Availability

Not applicable.

## Abbreviations

AASLD: American Association for the Study of Liver Diseases
APASL: Asian Pacific Association for the Study of the Liver
APHE: Arterial phase hyperenhancement
CE-CT: Contrast-enhanced computed tomography
EASL: European Association for the Study of the Liver
ECA-MRI: Extracellular contrast agent-enhanced magnetic resonance imaging
HBP: Hepatobiliary phase
HCC: Hepatocellular carcinoma
iRECIST: Immune-modified response evaluation criteria in solid tumors
JSH: Japan Society of Hepatology
KLCA–NCC: Korean Liver Cancer Association–National Cancer Center
LI-RADS: Liver Imaging Reporting and Data System
MRI: Magnetic resonance imaging
mRECIST: Modified Response Evaluation Criteria in Solid Tumors
OATP: Organic anion-transporting polypeptide
OPTN: Organ procurement and transplantation network
PVP: Portal venous phase
RECIST: Response evaluation criteria in solid tumors
T2WI: T2-weighted imaging
TACE: Transarterial chemoembolization
TARE: Transarterial radioembolization
TP: Transitional phase
TRA: Treatment response algorithm
SBRT: Stereotactic body radiation therapy
UNOS: United network for organ sharing
