# Supplementary material for: Hepatobiliary phase imaging in HCC: revisiting diagnostic algorithms and clinical utility of gadoxetic acid-enhanced MRI across international guidelines
Source: Insights Imaging. 2026 Sep 28;17:237. doi: 10.1186/s13244-026-02402-5 (PMC13620025; doi:10.1186/s13244-026-02402-5)
Supplement: Supplementary file 1 [file 13244_2026_2402_MOESM1_ESM.pdf]

# Hepatobiliary Phase Imaging in HCC: Revisiting Diagnostic Algorithms and Clinical Utility of Gadoxetic Acid-Enhanced MRI Across International Guidelines

## ELECTRONIC SUPPLEMENTARY MATERIAL

*Insights Imaging (2026) Zhou S, Yang J, Zhang S et al.*

**Supplementary Table 1.** Guideline-stratified Diagnostic Specificity of gadoxetic acid-enhanced MRI for Small HCC Lesions (≤30 mm): Comparison with CE-CT/ECA-MRI

| Studies | LI-RADS (v2018) | | EASL (v2018) | KLCA-NCC (v2018/2022) | APASL (v2017) |
|---|---|---|---|---|---|
| | *Without HBP /TP hypointensity* | *With HBP /TP hypointensity* | | | |
| ***Gadoxetic acid-enhanced MRI*** | | | | | |
| Lee et al.(2019)[1] (10 mm ≤ size ≤ 19 mm; n= 173) | 94 [89.1, 97.8] | | | | |
| Kim et al.(2019)[2] (TP) (0 mm < size ≤ 30 mm; n= 203) | 94 [67.6, 99.2] | 82 [56.5, 94.4] | | | |
| Kim et al.(2019)[2] (0 mm < size ≤ 30 mm; n= 203) | 94 [67.6, 99.2] | 82 [56.5, 94.4] | | | |
| Jiang et al.(2019)[3] (0 mm < size ≤ 20mm; n= 34) | 77 [46, 95] | | 69 [64, 97] | | |
| Hwang et al.(2019)[4](TP)[#] (0 mm < size < 20mm; n= N/A) | 96.7 [93.1, 100.0] | 93.9 [89.6, 98.2] | | | |
| Gaetano et al.(2019)[5] (0 mm < size ≤ 20mm; n= 43) | 76.9 [56.4-91.0] | | | | |
| Byun et al.(2020)[6] (10 mm ≤ size ≤ 30 mm; n=493) | 92.2 [80.9, 97.0] | | 84.3 [71.7, 92.0] | 78.4 [65.1, 87.6] | 70.6 [56.8, 81.4] |
| Kang et al.(2020)[7] (10 mm ≤ size ≤ 30 mm; n=385) | 95.1 [88.6–98.0] | | | | |
| Lee et al. (2020)[8] (10 mm ≤ size ≤ 19 mm; n= 104) | 97.6 [93.0, 100.0] | | | 95.2 [88.8, 100.0] | |
| Lee et al. (2020)[9] (10 mm ≤ size ≤ 19 mm; n= 85) | 100 [100, 100] | | 100 [100, 100] | | |
| Ko et al. (2020)[10] (10 mm ≤ size ≤ 19 mm; n= 90) | 90.3 [75.1, 96.6] | | | | |
| Jiang et al. (2021) [11] (0 mm < size ≤ 19 mm; n= 200) | 96.5 | | | | |
| Lee et al. (2021)[12] (10 mm ≤ size ≤ 19 mm; n= 173) | 89.1 [82.3–93.9] | | | 94.5 [89.1–97.8] | |
| Hwang et al. (2021)[13][#] (10 mm ≤ size ≤ 19 mm; n= N/A ) | 95.1 [90.2, 100.0] | | 95.1 [90.2, 100.0] | 86.1 [79.2, 93.0] | 86.1 [79.2, 93.0] |
| Park et al. (2021)[14][#] (10 mm ≤ size ≤ 30 mm; n=447) | 93.8 [91.3, 96.3] | | 94.5 [92.1, 96.9] | 87.6 [84.1, 91.1] | 82.5 [78.7, 86.3] |
| Park et al. (2021)[14][#] (10 mm ≤ size ≤ 19 mm; n= 249) | 95.5 [93.2, 97.8] | | 96.4 [94.2, 98.6] | 89.1 [85.5, 92.7] | 86.9 [83.2, 90.6] |
| Park et al. (2021)[14][#] (20 mm ≤ size ≤ 30 mm; n= 198) | 83.7 [73.8, 93.6] | | 83.7 [73.8, 93.6] | 79.4 [68.9, 89.9] | 56.1 [44.2, 68.0] |
| Clarke et al. (2021)[15] (10 mm ≤ size ≤ 19 mm; n= 74) | 93 | | 92 | | 86 |
| Zhang et al. (2021)[16] (10 mm ≤ size ≤ 19 mm; n= 65) | 96.2 | 96.2 | | | |
| Xie et al. (2022)[17] (TP) (10 mm ≤ size ≤ 19 mm; n= 174) | 98.7 [91.9-99.9] | 97.4 [90.0–99.5] | | | |
| Huang et al. (2023)[18][†] (0 mm < size < 10mm; n= 240) | 92.6 [85.5-96.5] | | | | 79.6 [70.6-86.5] |
| Yoon et al. (2024)[19] (10 mm ≤ size ≤ 19 mm; n= 766) | 91.6 [88.6, 93.9] | | 91.0 [87.9, 93.3] | 83.3 [79.6, 86.4] | 73.2 [68.7, 77.4] |
| Lin et al. (2025)[20] (0 mm < size < 30mm; n= 275) | 82.0* [68.6, 91.4] | 66.0* [51.2, 78.8] | | | |
| ***Gadoxetic acid-enhanced MRI vs. CE-CT*** | | | | | |
| Min et al.(2020) [21] ( size ≤ 19 mm; n= 84) | Equal 100.0 [88.6, 100.0] | | | | |
| *Yoon* et al. (2025)[22] (10 mm ≤ size ≤ 19 mm; n = 532) | Higher* 91.7 [87.8, 94.4] | | | Lower 84.6 [80.0, 88.3] | Lower* 76.0 [74.0, 89.0] |
| ***Gadoxetic acid-enhanced MRI vs. ECA-MRI*** | | | | | |
| Paisant et al.(2020)[23] (10 mm ≤ size ≤ 30 mm; n= 225) | | | Equal 83.3 72.7–91.1] | Lower* 68.1 [56.0, 78.6] | |
| Min et al.(2020) [21] ( size ≤ 19 mm; n= 84) | Equal 100.0 [88.6, 100.0] | | | | |
| Min et al. (2021)[24] (20 mm ≤ size ≤ 30 mm; n= 101) | Equal 78.6 [50.6-92.9] | | Equal 78.6 [50.6-92.9] | | |
| Min et al. (2021)[24] (10 mm ≤ size ≤ 19 mm; n= 74) | Equal 100 [86.8-100] | | Equal 100 [86.8-100] | | |
| Yoon et al. (2023)[25] (10 mm ≤ size ≤ 19 mm; n= 53) | | | | Equal 95.0 [71.8-99.3] | |

N, number of lesions; ECA-MRI, extracellular contrast agent–enhanced magnetic resonance imaging; CE-CT, contrast-enhanced computed tomography; TP, transitional phase; LI-RADS, Liver Imaging Reporting and Data System; EASL, European Association for the Study of the Liver; KLCA-NCC, Korean Liver Cancer Association National Cancer Center; APASL, Asian Pacific Association for the Study of the Liver.

"(TP)" indicates washout assessed on the transitional phase; otherwise, on the hepatobiliary phase.

# Results from multiple readers were pooled using a fixed-effect model. When calculation of a weighted average was not feasible, the most representative result was reported.

* Statistically significant ($P < 0.05$).

† For lesions <1 mm, LI-RADS categorizes them as LR-4.

**Supplementary Table 2.** Guideline-Stratified Diagnostic Sensitivity of gadoxetic acid-enhanced MRI for HCC Lesions of All Sizes: Comparison with CE-CT/ECA-MRI

| **Studies** | **LI-RADS (v2018)** | | **EASL (v2018)** | **KLCA-NCC (v2018/2022)** | **APASL (v2017)** |
|---|---|---|---|---|---|
| | *Without HBP /TP hypointensity* | *With HBP /TP hypointensity* | | | |
| ***Gadoxetic acid-enhanced MRI*** | | | | | |
| Min et al. (2018)[26] (TP)<br>(size > 0 mm; n= 117) | 66.3* | 80.0* | | | |
| Kim et al.(2019)[27]<br>(50mm ≥ size > 0mm; n= 368) | 55.9<br>[49.8–61.9] | | | | |
| Lee et al.(2019)[1]<br>(size > 10 mm; n = 422) | 81.0<br>[75.1, 85.6] | | | | |
| Jiang et al.(2019)[3]<br>(size > 0 mm; n= 229) | 86<br>[80, 91] | | 91<br>[85, 95] | | |
| Jiang et al.(2019)[3]<br>(50mm ≥ size > 20mm; n= 83) | 80<br>[68, 89] | | 85<br>[74, 93] | | |
| Jiang et al.(2019)[3]<br>(size > 50mm; n= 112) | 92<br>[85, 97] | | 96<br>[89, 99] | | |
| Hwang et al.(2019)[4] (TP)[#]<br>(size > 0 mm; n= 214) | 61.2*<br>[55.7, 66.7] | 77.5*<br>[72.8, 82.2] | | | |
| Hwang et al.(2019)[4] (TP)[#]<br>(size ≥ 20mm; n= N/A) | 70.6*<br>[64.4, 76.8] | 84.9*<br>[79.8, 90.0] | | | |
| Kim et al. (2020)[28]<br>(size > 0 mm; n= 165) | 73.5 | | | | |
| Jeon et al. (2020)[29]<br>(size > 0 mm; n= 154) | 34.5<br>[26.5, 43.4] | | 38.8<br>[30.2, 48.2] | 65.5<br>[54.5, 75.1] | 75.9<br>[65.2, 84.1] |
| Jeon et al. (2020)[29]<br>(size > 10 mm; n= 137) | 37.4<br>[28.5, 47.2] | 71.0<br>[60.1, 80.0] | 42.1<br>[32.0, 52.9] | 71.0<br>[60.1, 80.0] | 75.7<br>[64.4, 84.3] |
| Jeon et al. (2020)[29]<br>(size > 20 mm; n= 54) | 51.1<br>[35.7, 66.2] | | 55.3<br>[39.0, 70.5] | 78.7<br>[64.6, 88.3] | 83.0<br>[69.0, 91.4] |
| Lee et al. (2020)[8]<br>(size ≥ 10mm; n= 266) | 68.2<br>[61.7, 74.6] | | | 79.1<br>[73.5, 84.7] | |
| Lee et al. (2020)[8]<br>(size ≥ 20mm; n= 162) | 75.5<br>[68.4, 82.7] | | | 80.6<br>[74.0, 87.2] | |
| Lee et al. (2020)[9]<br>(size > 10 mm; n= 218) | 67.5<br>[60.3, 74.7] | | 67.5<br>[60.3, 74.7] | | |
| Lee et al. (2020)[9]<br>(size ≥ 20 mm; n= 133) | 76.1<br>[68.2, 84.0] | | 76.1<br>[68.2, 84.0] | | |
| Cha et al. (2020)[30]<br>(50mm ≥ size ≥ 10mm; n= 147) | 59.9*<br>[50.9–68.3] | 74.9*<br>[66.4, 81.8] | | | |
| Park et al. (2020)[31] (TP)<br>(size > 0 mm; n= 792) | 71.9*<br>[67.7, 75.7] | 75.6*<br>[71.6, 79.3] | | | |
| Ko et al. (2020)[10]<br>(size ≥ 10mm; n= 137) | 58.4<br>[48.1, 68.1] | | | | |
| Ko et al. (2020)[10]<br>(size ≥ 20mm; n= 47) | 70.0<br>[52.1, 83.3] | | | | |
| Jiang et al. (2021) [11]<br>(size > 0 mm; n= 568) | 60.7 | | | | |
| Jiang et al. (2021) [11]<br>(size > 20 mm; n=175) | 90.8 | | | | |
| Lee et al. (2021)[12]<br>(size ≥ 10mm; n= 422) | 87.2<br>[82.2–91.2] | | | 80.8<br>[75.1–85.6] | |
| Lee et al. (2021)[12]<br>(size ≥ 20mm; n= 249) | 86.8<br>[81.1–91.3] | | | 82.0<br>[75.8–87.2] | |
| Lee et al. (2021)[32]<br>(size ≥ 10mm; n= 366) | 66.2*<br>[60.3–71.7] | 76.5*<br>[71.1–81.3] | | | |
| Hwang et al. (2021)[13][#]<br>(size > 0 mm; n= 149) | 62.7<br>[57.2, 68.2] | | 54.0<br>[48.3, 59.7] | 85.0<br>[80.9, 89.1] | 92.0<br>[88.8, 95.2] |
| Clarke et al. (2021)[15]<br>(size ≥ 10mm; n= 105) | 45 | | 44 | | 64 |
| Zhang et al. (2021)[16]<br>(size ≥ 0 mm; n= 250) | 78.1* | 82.7* | | | |
| Zhang et al. (2021)[16]<br>(size ≥ 20 mm; n= 185) | 81.5 | 83.4 | | | |
| Kang et al. (2021)[33]<br>(size ≥ 10mm; n= 160) | 50.0<br>[40.6, 89.4] | | 53.5<br>[44.0, 62.8] | | |
| Odedra et al.(2022)[34][#]<br>(size ≥ 10 mm; n= 72) | 35.7 | | 32.1 | 60.7 | 59.5 |
| Odedra et al.(2022)[34][#]<br>(size > 0 mm; n= 115) | 29.1 | | 26.2 | 49.5 | 63.1 |
| Yoon et al. (2024) [19]<br>(size ≥ 10mm; n = 2445) | 70.5<br>[68.8, 72.3] | | 69.3<br>[67.5, 71.1] | 78.2<br>[76.6, 79.7] | 89.1<br>[87.8, 90.3] |
| Hu et al. (2024)[35] (TP)<br>(size > 0 mm; n= 426) | 74.4* | 80.0* | | | |
| Hu et al. (2024)[35]<br>(size > 0 mm; n= 426) | 74.4* | 80.8* | | | |
| Li et al. (2024)[36]<br>(size > 0 mm; n= 445) | 82.4<br>[78.1, 85.9] | | | | |

N, number of lesions; ECA-MRI, extracellular contrast agent–enhanced magnetic resonance imaging; CE-CT, contrast-enhanced computed tomography; TP, transitional phase; LI-RADS, Liver Imaging Reporting and Data System; EASL, European Association

for the Study of the Liver; KLCA-NCC, Korean Liver Cancer Association National Cancer Center; APASL, Asian Pacific Association for the Study of the Liver.
"(TP)" indicates washout assessed on the transitional phase; otherwise, on the hepatobiliary phase.
# Results from multiple readers were pooled using a fixed-effect model. When calculation of a weighted average was not feasible, the most representative result was reported.
* Statistically significant ($P < 0.05$).

**Supplementary Table 2.** Guideline-Stratified Diagnostic Sensitivity of gadoxetic acid-enhanced MRI for HCC Lesions of All Sizes: Comparison with CE-CT/ECA-MRI (continue)

| Studies | LI-RADS (v2018) | | EASL (v2018) | KLCA-NCC (v2018/2022) | APASL (v2017) |
|---|---|---|---|---|---|
| | *Without HBP /TP hypointensity* | *With HBP /TP hypointensity* | | | |
| ***Gadoxetic acid-enhanced MRI vs. CE-CT*** | | | | | |
| Yoon et al.(2019)[37] (size ≥ 20 mm; n= 7) | Higher 40.0 [5.3, 85.4] | | | | |
| Nakao et al.(2020)[38] (size > 0 mm; n= 110) | Lower 37.7% | | | | |
| Min et al.(2020) [21] (size > 0 mm; n= 163) | Higher 71.2 [62.4, 78.6] | | | | |
| Min et al.(2020) [21] (size ≥ 20 mm; n= 79) | Higher 75.7 [63.2, 85.0] | | | | |
| Chen et al.(2020)[39] (size ≥ 10 mm; n= 108) | Higher* 86.9 | | | | |
| *Yoon* et al. (2025)[22] (size ≥ 10mm; n = 1590) | Lower* 70.6 [68.4, 72.6] | | | Higher* 78.7 [76.7, 85.0] | Higher* 89.3 [87.7, 90.8] |
| ***Gadoxetic acid-enhanced MRI vs. ECA-MRI*** | | | | | |
| Min et al. (2018)[26] (size > 0 mm; n= 117) | Lower* 66.3 | | | | |
| Kim et al.(2020) [40] (TP) (size ≥ 20; n= 66) | Lower 83.3 | Higher 92.4 | | | |
| Kim et al.(2020) [40] (TP) (size > 0 mm; n= 114) | Higher 78.1 | Higher* 91.2 | | | |
| Cha et al. (2020)[30] (50mm ≥ size ≥ 10mm; n= 147) | Lower* 59.9 [50.9–68.3] | Lower 74.9 [66.4–81.8] | | | |
| Min et al.(2020) [21] (size > 0 mm; n= 163) | Lower* 71.2 [62.4, 78.6] | | | | |
| Min et al.(2020) [21] (size ≥ 20 mm; n= 79) | Lower* 75.7 [63.2, 85.0] | | | | |
| Lee et al. (2020)[9] (size > 10 mm; n= 218) | Lower* 67.5 [60.3, 74.7] | | Lower 67.5 [60.3, 74.7] | | |
| Min et al. (2021)[24] (size > 10 mm; n= 215) | Lower* 67.3 [60.0-73.8] | | Lower* 63.0 [55.6-69.9] | | |
| Yoon et al. (2023)[25] (size ≥ 10mm; n= 118) | | | | Higher* 78.5 [69.0-85.7] | |
| Yoon et al. (2023)[25] (size ≥ 20mm; n= 65) | | | | Higher* 81.7 [69.8-89.5] | |
| Yoon et al. (2023)[41] (size ≥ 10mm; n= 345) | | | | Higher* 77.0 [71.5–81.9] | |

N, number of lesions; ECA-MRI, extracellular contrast agent–enhanced magnetic resonance imaging; CE-CT, contrast-enhanced computed tomography; TP, transitional phase; HBP, hepatobiliary phase; LI-RADS, Liver Imaging Reporting and Data System; EASL, European Association for the Study of the Liver; KLCA-NCC, Korean Liver Cancer Association National Cancer Center; APASL, Asian Pacific Association for the Study of the Liver.
"(TP)" indicates washout assessed on the transitional phase; otherwise, on the hepatobiliary phase.
* Statistically significant ($P < 0.05$).

**Supplementary Table 3.** Guideline-Stratified Diagnostic Specificity of gadoxetic acid-enhanced MRI for HCC Lesions of All Sizes: Comparison with CE-CT/ECA-MRI

| Studies | LI-RADS (v2018) | | EASL (v2018) | KLCA-NCC (v2018/2022) | APASL (v2017) |
|---|---|---|---|---|---|
| | *Without HBP /TP hypointensity* | *With HBP /TP hypointensity* | | | |
| ***Gadoxetic acid-enhanced MRI*** | | | | | |
| Min et al. (2018)[26] (TP)<br>(size > 0 mm; n= 117) | 100 | 100 | | | |
| Kim et al.(2019)[27]<br>(50mm ≥ size > 0mm; n= 368) | 94.9<br>[88.1–97.9] | | | | |
| Lee et al.(2019)[1]<br>(size > 10 mm; n = 422) | 91<br>[85.9, 94.6] | | | | |
| Jiang et al.(2019)[3]<br>(size > 0 mm; n= 229) | 82<br>[70, 91] | | 71<br>[58, 83] | | |
| Jiang et al.(2019)[3]<br>(50mm ≥ size > 20mm; n= 83) | 91<br>[71, 99] | | 82<br>[60, 95] | | |
| Jiang et al.(2019)[3]<br>(size > 50mm; n= 112) | 76<br>[53, 92] | | 62<br>[38, 82] | | |
| Hwang et al.(2019)[4] (TP)[#]<br>(size > 0 mm; n= 214) | 94.8<br>[91.3, 98.3] | 91.4<br>[87.2, 95.6] | | | |
| Hwang et al.(2019)[4] (TP)[#]<br>(size ≥ 20mm; n= N/A) | 87.1<br>[75.6, 98.6] | 81.7<br>[69.3, 94.1] | | | |
| Kim et al. (2020)[28]<br>(size > 0 mm; n= 165) | 76.9 | | | | |
| Jeon et al. (2020)[29]<br>(size > 0 mm; n= 154) | 97.4<br>[83.1, 99.6] | | 92.1<br>[77.3, 97.6] | 92.1<br>[77.8, 97.5] | 78.9<br>[62.0, 89.6] |
| Jeon et al. (2020)[29]<br>(size > 10 mm; n= 137) | 96.7<br>[79.5, 99.5] | | 90.0<br>[72.5, 96.9] | 90.0<br>[72.5, 96.9] | 80.0<br>[60.7, 91.2] |
| Jeon et al. (2020)[29]<br>(size > 20 mm; n= 54) | 85.7<br>[41.9, 98.0] | | 71.4<br>[32.7, 92.8] | 85.7<br>[41.9, 98.0] | 57.1<br>[23.0, 85.6] |
| Lee et al. (2020)[8]<br>(size ≥ 10mm; n= 266) | 95.4<br>[90.3, 100.0] | | | 93.9<br>[88.0, 99.7] | |
| Lee et al. (2020)[8]<br>(size ≥ 20mm; n= 162) | 91.3<br>[79.8, 100.0] | | | 91.3<br>[79.8, 100.0] | |
| Lee et al. (2020)[9]<br>(size > 10 mm; n= 218) | 98.0<br>[94.7, 100] | | 96.4<br>[91.4, 100] | | |
| Lee et al. (2020)[9]<br>(size ≥ 20 mm; n= 133) | 95.0<br>[85.5, 100] | | 90.0<br>[76.9, 100] | | |
| Cha et al. (2020)[30]<br>(50mm ≥ size ≥ 10mm; n= 147) | 100<br>[0.87, 1.00) | 88.0<br>[0.70, 0.96] | | | |
| Park et al. (2020)[31] (TP)<br>(size > 0 mm; n= 792) | 94.4<br>[91.0, 96.8] | 96.8<br>[94.1, 98.5] | | | |
| Ko et al. (2020)[10]<br>(size ≥ 10mm; n= 137) | 85.4<br>[72.8, 92.6] | | | | |
| Ko et al. (2020)[10]<br>(size ≥ 20mm; n= 47) | 76.5<br>[52.7, 90.5] | | | | |
| Jiang et al. (2021) [11]<br>(size > 0 mm; n= 568) | 94.2 | | | | |
| Jiang et al. (2021) [11]<br>(size > 20 mm; n=175) | 56.5 | | | | |
| Lee et al. (2021)[12]<br>(size ≥ 10mm; n= 422) | 86.2<br>[80.4–90.8] | | | 91.0<br>[85.9–94.6] | |
| Lee et al. (2021)[12]<br>(size ≥ 20mm; n= 249) | 80.0<br>[67.7–89.2] | | | 83.3<br>[71.5–91.7] | |
| Lee et al. (2021)[32]<br>(size ≥ 10mm; n= 366) | 96.5<br>[90.0–99.3] | 95.3<br>[88.4–98.7] | | | |
| Hwang et al. (2021)[13][#]<br>(size > 0 mm; n= 149) | 93.7<br>[90.0, 97.4] | | 92.6<br>[88.5, 96.7] | 86.7<br>[81.8, 91.6] | 75.6<br>[69.4, 81.8] |
| Clarke et al. (2021)[15]<br>(size ≥ 10mm; n= 105) | 89 | | 86 | | 81 |
| Zhang et al. (2021)[16]<br>(size ≥ 0 mm; n= 250) | 96.3 | 96.3 | | | |
| Zhang et al. (2021)[16]<br>(size ≥ 20 mm; n= 185) | 96.4 | 96.4 | | | |
| Kang et al. (2021)[33]<br>(size ≥ 10mm; n= 160) | 93.2<br>[81.3, 98.6] | | 93.2<br>[81.3, 98.6] | | |
| Odedra et al.(2022)[34][#]<br>(size ≥ 10 mm; n= 72) | 100.0 | | 100.0 | 91.7 | 100.0 |
| Odedra et al.(2022)[34][#]<br>(size > 0 mm; n= 115) | 100.0 | | 100.0 | 91.7 | 100.0 |
| Yoon et al. (2024)[19]<br>(size ≥ 10mm; n = 2445) | 89.6<br>[87.8, 91.2] | | 88.1<br>[86.2, 89.9] | 84.1<br>[81.9, 86.0] | 52.2<br>[49.0, 55.4] |
| Hu et al. (2024)[35] (TP)<br>(size > 0 mm; n= 426) | 88.6 | 86.9 | | | |
| Hu et al. (2024)[35]<br>(size > 0 mm; n= 426) | 88.6 | 85.8 | | | |
| Li et al. (2024)[36]<br>(size > 0 mm; n= 445) | 90.7<br>[78.9, 96.5] | | | | |

ECA-MRI, extracellular contrast agent–enhanced magnetic resonance imaging; CE-CT, contrast-enhanced computed tomography; TP, transitional phase; HBP, hepatobiliary phase; LI-RADS, Liver Imaging Reporting and Data System; EASL, European Association for

the Study of the Liver; KLCA-NCC, Korean Liver Cancer Association National Cancer Center; APASL, Asian Pacific Association for the Study of the Liver
"(TP)" indicates washout assessed on the transitional phase; otherwise, on the hepatobiliary phase.
# Results from multiple readers were pooled using a fixed-effect model. When calculation of a weighted average was not feasible, the most representative result was reported.
* Statistically significant ($P < 0.05$).

**Supplementary Table 3.** Guideline-Stratified Diagnostic Specificity of gadoxetic acid-enhanced MRI for HCC Lesions of All Sizes: Comparison with CE-CT/ECA-MRI (continue)

| **Studies** | **LI-RADS (v2018)** | | **EASL (v2018)** | **KLCA-NCC (v2018/2022)** | **APASL (v2017)** |
|---|---|---|---|---|---|
| | *Without HBP /TP hypointensity* | *With HBP /TP hypointensity* | | | |
| ***Gadoxetic acid-enhanced MRI vs. CECT*** | | | | | |
| Yoon et al.(2019)[37] (size ≥ 20 mm; n= 7) | Equal 100 [15.8, 100] | | | | |
| Min et al.(2020) [21] (size > 0 mm; n= 163) | Equal 97.4 [84.6, 99.6] | | | | |
| Min et al.(2020) [21] (size ≥ 20 mm; n= 79) | Equal 88.9 [50.0, 98.5] | | | | |
| Chen et al.(2020)[39] (size ≥ 10 mm; n= 108) | Equal 88.9 | | | | |
| *Yoon* et al. (2025)[22] (size ≥ 10mm; n = 1590) | Higher* 90.4 [88.1, 92.3] | | | Equal 84.9 [82.1, 87.4] | Lower* 54.3 [50.0, 58.5] |
| ***Gadoxetic acid-enhanced MRI vs. ECA-MRI*** | | | | | |
| Min et al. (2018)[26] (size > 0 mm; n= 117) | Equal 100 | | | | |
| Cha et al. (2020)[30] (50mm ≥ size ≥ 10mm; n= 147) | Equal 100 [0.87, 1.00] | Lower 88.0 [0.70, 0.96] | | | |
| Min et al.(2020) [21] (size > 0 mm; n= 163) | Equal 97.4 [84.6, 99.6] | | | | |
| Min et al.(2020) [21] (size ≥ 20 mm; n= 79) | Equal 88.9 [50.0, 98.5] | | | | |
| Lee et al. (2020)[9] (size > 10 mm; n= 218) | Higher 98.2 [94.7, 100] | | Higher 96.4 [91.4, 100] | | |
| Min et al. (2021)[24] (size > 10 mm; n= 215) | Higher 91.5 [77.0-97.2] | | Higher 91.5 [77.0-97.2] | | |
| Yoon et al. (2023)[25] (size ≥ 10mm; n= 118) | | | | Equal 92.0 [73.1-98.0] | |
| Yoon et al. (2023)[25] (size ≥ 20mm; n= 65) | | | | Equal 80.0 [30.9-97.3] | |
| Yoon et al. (2023)[41] (size ≥ 10mm; n= 345) | | | | Lower 94.7 (87.1–98.6) | |

ECA-MRI, extracellular contrast agent–enhanced magnetic resonance imaging; CE-CT, contrast-enhanced computed tomography; LI-RADS, Liver Imaging Reporting and Data System; EASL, European Association for the Study of the Liver; KLCA-NCC, Korean Liver Cancer Association‐National Cancer Center; APASL, Asian Pacific Association for the Study of the Liver

# Results from multiple readers were pooled using a fixed-effect model. When calculation of a weighted average was not feasible, the most representative result was reported.

* Statistically significant ($P < 0.05$).

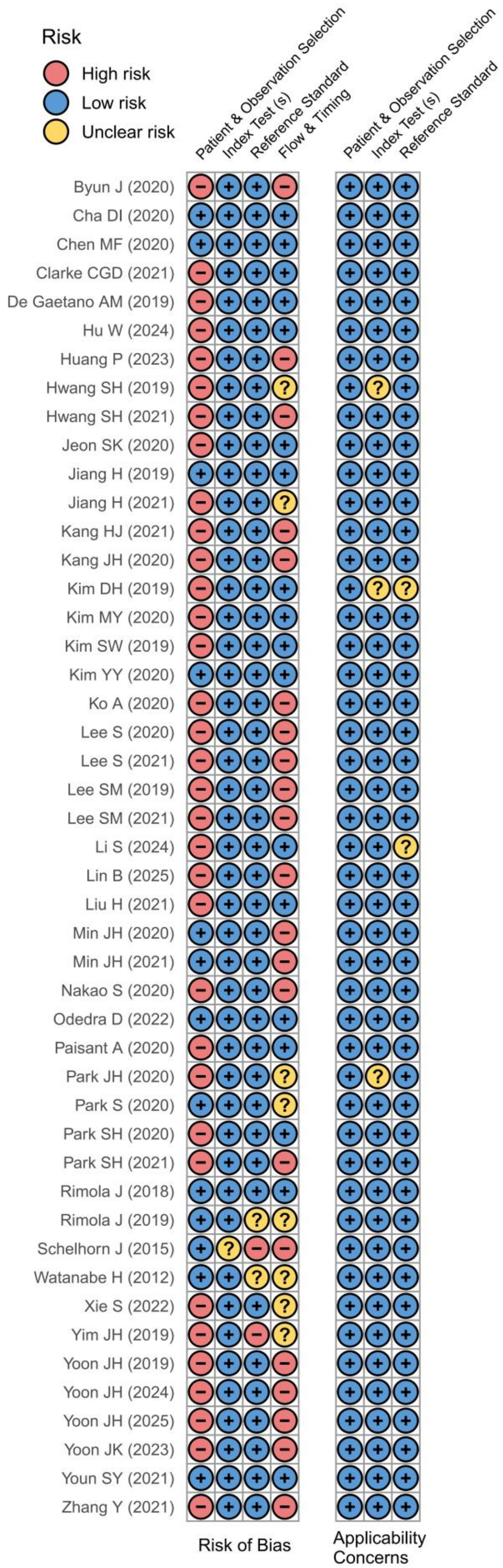


**Supplementary Figure 1:** Quality assessment of included diagnostic accuracy studies using the Quality Assessment of Diagnostic Accuracy Studies-2 (QUADAS-2) tool. Risk of bias and

applicability concerns were evaluated across the four standard domains: patient selection, index test, reference standard, and flow and timing.

**References**

[1] Lee S M, Lee J M, Ahn S J, Kang H J, Yang H K, Yoon J H. LI-RADS Version 2017 versus Version 2018: Diagnosis of Hepatocellular Carcinoma on Gadoxetate Disodium-enhanced MRI. Radiology, 2019, 292(3): 655-663.http://dx.doi.org/10.1148/radiol.2019182867

[2] Kim D H, Choi S H, Kim S Y, Kim M J, Lee S S, Byun J H. Gadoxetic Acid-enhanced MRI of Hepatocellular Carcinoma: Value of Washout in Transitional and Hepatobiliary Phases. Radiology, 2019, 291(3): 651-657.http://dx.doi.org/10.1148/radiol.2019182587

[3] Jiang H, Liu X, Chen J, Wei Y, Lee J M, Cao L, et al. Man or machine? Prospective comparison of the version 2018 EASL, LI-RADS criteria and a radiomics model to diagnose hepatocellular carcinoma. Cancer Imaging, 2019, 19(1): 84.http://dx.doi.org/10.1186/s40644-019-0266-9

[4] Hwang S H, Park S, Han K, Choi J Y, Park Y N, Park M S. Optimal lexicon of gadoxetic acid-enhanced magnetic resonance imaging for the diagnosis of hepatocellular carcinoma modified from LI-RADS. Abdom Radiol (NY), 2019, 44(9): 3078-3088.http://dx.doi.org/10.1007/s00261-019-02077-1

[5] De Gaetano A M, Catalano M, Pompili M, Marini M G, Rodríguez Carnero P, Gullì C, et al. Critical analysis of major and ancillary features of LI-RADS v2018 in the differentiation of small (≤ 2 cm) hepatocellular carcinoma from dysplastic nodules with gadobenate dimeglumine-enhanced magnetic resonance imaging. Eur Rev Med Pharmacol Sci, 2019, 23(18): 7786-7801.http://dx.doi.org/10.26355/eurrev_201909_18988

[6] Byun J, Choi S H, Byun J H, Lee S J, Kim S Y, Won H J, et al. Comparison of the diagnostic performance of imaging criteria for HCCs ≤ 3.0 cm on gadoxetate disodium-enhanced MRI. Hepatol Int, 2020, 14(4): 534-543.http://dx.doi.org/10.1007/s12072-020-10040-2

[7] Kang J H, Choi S H, Byun J H, Kim D H, Lee S J, Kim S Y, et al. Ancillary features in the Liver Imaging Reporting and Data System: how to improve diagnosis of hepatocellular carcinoma ≤ 3 cm on magnetic resonance imaging. Eur Radiol, 2020, 30(5): 2881-2889.http://dx.doi.org/10.1007/s00330-019-06645-3

[8] Lee S, Kim S S, Chang D R, Kim H, Kim M J. Comparison of LI-RADS 2018 and KLCA-NCC 2018 for noninvasive diagnosis of hepatocellular carcinoma using magnetic resonance imaging. Clin Mol Hepatol, 2020, 26(3): 340-351.http://dx.doi.org/10.3350/cmh.2020.0004

[9] Lee S, Kim M J, Kim S S, Shin H, Kim D Y, Choi J Y, et al. Retrospective comparison of EASL 2018 and LI-RADS 2018 for the noninvasive diagnosis of hepatocellular carcinoma using magnetic resonance imaging. Hepatol Int, 2020, 14(1): 70-79.http://dx.doi.org/10.1007/s12072-019-10002-3

[10] Ko A, Park H J, Lee E S, Park S B, Kim Y K, Choi S Y, et al. Comparison of the diagnostic performance of the 2017 and 2018 versions of LI-RADS for hepatocellular carcinoma on

gadoxetic acid enhanced MRI. Clin Radiol, 2020, 75(4): 319.e1-319.e9.http://dx.doi.org/10.1016/j.crad.2019.11.004

[11] Jiang H, Song B, Qin Y, Konanur M, Wu Y, Mcinnes M D F, et al. Modifying LI-RADS on Gadoxetate Disodium-Enhanced MRI: A Secondary Analysis of a Prospective Observational Study. J Magn Reson Imaging, 2022, 56(2): 399-412.http://dx.doi.org/10.1002/jmri.28056

[12] Lee S M, Lee J M, Ahn S J, Kang H J, Yang H K, Yoon J H. Diagnostic Performance of 2018 KLCA-NCC Practice Guideline for Hepatocellular Carcinoma on Gadoxetic Acid-Enhanced MRI in Patients with Chronic Hepatitis B or Cirrhosis: Comparison with LI-RADS Version 2018. Korean J Radiol, 2021, 22(7): 1066-1076.http://dx.doi.org/10.3348/kjr.2020.0846

[13] Hwang S H, Park M S, Park S, Lim J S, Kim S U, Park Y N. Comparison of the current guidelines for diagnosing hepatocellular carcinoma using gadoxetic acid-enhanced magnetic resonance imaging. Eur Radiol, 2021, 31(7): 4492-4503.http://dx.doi.org/10.1007/s00330-020-07468-3

[14] Park S H, Shim Y S, Kim B, Kim S Y, Kim Y S, Huh J, et al. Retrospective analysis of current guidelines for hepatocellular carcinoma diagnosis on gadoxetic acid-enhanced MRI in at-risk patients. Eur Radiol, 2021, 31(7): 4751-4763.http://dx.doi.org/10.1007/s00330-020-07577-z

[15] Clarke C G D, Albazaz R, Smith C R, Rowe I, Treanor D, Wyatt J I, et al. Comparison of LI-RADS with other non-invasive liver MRI criteria and radiological opinion for diagnosing hepatocellular carcinoma in cirrhotic livers using gadoxetic acid with histopathological explant correlation. Clin Radiol, 2021, 76(5): 333-341.http://dx.doi.org/10.1016/j.crad.2020.12.007

[16] Zhang Y, Tang W, Xie S, Chen J, Zhang L, Rong D, et al. The role of lesion hypointensity on gadobenate dimeglumine-enhanced hepatobiliary phase MRI as an additional major imaging feature for HCC classification using LI-RADS v2018 criteria. Eur Radiol, 2021, 31(10): 7715-7724.http://dx.doi.org/10.1007/s00330-021-07807-y

[17] Xie S, Zhang Y, Chen J, Jiang T, Liu W, Rong D, et al. Can modified LI-RADS increase the sensitivity of LI-RADS v2018 for the diagnosis of 10-19 mm hepatocellular carcinoma on gadoxetic acid-enhanced MRI? Abdom Radiol (NY), 2022, 47(2): 596-607.http://dx.doi.org/10.1007/s00261-021-03339-7

[18] Huang P, Zhou C, Wu F, Xiao Y, Qian X, Wang Y, et al. An improved diagnostic algorithm for subcentimeter hepatocellular carcinoma on gadoxetic acid-enhanced MRI. Eur Radiol, 2023, 33(4): 2735-2745.http://dx.doi.org/10.1007/s00330-022-09282-5

[19] Yoon J H, Kim Y K, Kim J W, Chang W, Choi J I, Park B J, et al. Comparison of Four Diagnostic Guidelines for Hepatocellular Carcinoma Using Gadoxetic Acid-enhanced Liver MRI. Radiology, 2024, 311(1): e233114.http://dx.doi.org/10.1148/radiol.233114

[20] Lin B, Zhang W, Jiang Y, Qin Y, Mishra P K, Chen J Y, et al. Diagnostic performance of LR-5 based on hypointensity on Gd-EOB-DTPA-enhanced MRI in the hepatobiliary phase for sHCC using LI-RADS v2018 criteria. Clin Radiol, 2025, 81: 106784.http://dx.doi.org/10.1016/j.crad.2024.106784

[21] Min J H, Kim J M, Kim Y K, Cha D I, Kang T W, Kim H, et al. Magnetic Resonance Imaging With Extracellular Contrast Detects Hepatocellular Carcinoma With Greater Accuracy Than With Gadoxetic Acid or Computed Tomography. Clin Gastroenterol Hepatol, 2020, 18(9): 2091-2100.e7.http://dx.doi.org/10.1016/j.cgh.2019.12.010

[22] Yoon J H, Chang W, Kim Y K, Lee C H, Kim J W, Park B J, et al. Comparison of Gadoxetic Acid-Enhanced Liver Magnetic Resonance Imaging and Contrast-Enhanced Computed Tomography for the Noninvasive Diagnosis of Hepatocellular Carcinoma. Liver Cancer, 2025: 1-13.http://dx.doi.org/10.1159/000545965

[23] Paisant A, Vilgrain V, Riou J, Oberti F, Sutter O, Laurent V, et al. Comparison of extracellular and hepatobiliary MR contrast agents for the diagnosis of small HCCs. J Hepatol, 2020, 72(5): 937-945.http://dx.doi.org/10.1016/j.jhep.2019.12.011

[24] Min J H, Kim J M, Kim Y K, Kim H, Cha D I, Kang T W, et al. EASL versus LI-RADS: Intra-individual comparison of MRI with extracellular contrast and gadoxetic acid for diagnosis of small HCC. Liver Int, 2021, 41(12): 2986-2996.http://dx.doi.org/10.1111/liv.15012

[25] Yoon J K, Han D H, Lee S, Choi J Y, Choi G H, Kim D Y, et al. Intraindividual Comparison of MRIs with Extracellular and Hepatobiliary Contrast Agents for the Noninvasive Diagnosis of Hepatocellular Carcinoma Using the Korean Liver Cancer Association-National Cancer Center 2022 Criteria. Cancer Res Treat, 2023, 55(3): 939-947.http://dx.doi.org/10.4143/crt.2022.1645

[26] Min J H, Kim J M, Kim Y K, Kang T W, Lee S J, Choi G S, et al. Prospective Intraindividual Comparison of Magnetic Resonance Imaging With Gadoxetic Acid and Extracellular Contrast for Diagnosis of Hepatocellular Carcinomas Using the Liver Imaging Reporting and Data System. Hepatology, 2018, 68(6): 2254-2266.http://dx.doi.org/10.1002/hep.30122

[27] Kim D H, Choi S H, Byun J H, Kang J H, Lim Y S, Lee S J, et al. Arterial subtraction images of gadoxetate-enhanced MRI improve diagnosis of early-stage hepatocellular carcinoma. J Hepatol, 2019, 71(3): 534-542.http://dx.doi.org/10.1016/j.jhep.2019.05.005

[28] Kim M Y, Joo I, Kang H J, Bae J S, Jeon S K, Lee J M. LI-RADS M (LR-M) criteria and reporting algorithm of v2018: diagnostic values in the assessment of primary liver cancers on gadoxetic acid-enhanced MRI. Abdom Radiol (NY), 2020, 45(8): 2440-2448.http://dx.doi.org/10.1007/s00261-020-02545-z

[29] Jeon S K, Lee J M, Joo I, Yoo J, Park J Y. Comparison of guidelines for diagnosis of hepatocellular carcinoma using gadoxetic acid-enhanced MRI in transplantation candidates. Eur Radiol, 2020, 30(9): 4762-4771.http://dx.doi.org/10.1007/s00330-020-06881-y

[30] Cha D I, Choi G S, Kim Y K, Kim J M, Kang T W, Song K D, et al. Extracellular contrast-enhanced MRI with diffusion-weighted imaging for HCC diagnosis: prospective comparison with gadoxetic acid using LI-RADS. Eur Radiol, 2020, 30(7): 3723-3734.http://dx.doi.org/10.1007/s00330-020-06753-5

[31] Park J H, Chung Y E, Seo N, Choi J Y, Park M S, Kim M J. Gadoxetic acid-enhanced MRI of hepatocellular carcinoma: Diagnostic performance of category-adjusted LR-5 using modified criteria. PLoS One, 2020, 15(11): e0242344.http://dx.doi.org/10.1371/journal.pone.0242344

[32] Lee S, Kim S S, Bae H, Shin J, Yoon J K, Kim M J. Application of Liver Imaging Reporting and Data System version 2018 ancillary features to upgrade from LR-4 to LR-5 on gadoxetic acid-enhanced MRI. Eur Radiol, 2021, 31(2): 855-863.http://dx.doi.org/10.1007/s00330-020-07146-4

[33] Kang H J, Lee J M, Jeon S K, Jang S, Park S, Joo I, et al. Intra-individual comparison of dual portal venous phases for non-invasive diagnosis of hepatocellular carcinoma at gadoxetic acid-enhanced liver MRI. Eur Radiol, 2021, 31(2): 824-833.http://dx.doi.org/10.1007/s00330-020-07162-4

[34] Odedra D, Jandaghi A B, Bhayana R, Elbanna K Y, Espin-Garcia O, Fischer S E, et al. Comparison of international guidelines for diagnosis of hepatocellular carcinoma and implications for transplant allocation in liver transplantation candidates with gadoxetic acid enhanced liver MRI versus contrast enhanced CT: a prospective study with liver explant histopathological correlation. Cancer Imaging, 2022, 22(1): 55.http://dx.doi.org/10.1186/s40644-022-00497-9

[35] Hu W, Lyu R, Wang D, Gao Z, Sun C, Jia K. Liver imaging reporting and data system diagnostic performance in hepatocellular carcinoma when modifying the definition of "washout" on gadoxetic acid-enhanced magnetic resonance imaging. Arab J Gastroenterol, 2024, 25(1): 58-63.http://dx.doi.org/10.1016/j.ajg.2023.12.012

[36] Li S, Deng K, Qiu J, Wang P, Yin D, Xie Y, et al. Based on Gadolinium Ethoxybenzyl DTPA-Enhanced MRI: Diagnostic Performance of the Category-Modified LR-5 Criteria in Patients At Risk for Hepatocellular Carcinoma. Technol Cancer Res Treat, 2024, 23: 15330338241256859.http://dx.doi.org/10.1177/15330338241256859

[37] Yoon J H, Lee J M, Lee Y J, Lee K B, Han J K. Added Value of sequentially performed gadoxetic acid-enhanced liver MRI for the diagnosis of small (10-19 mm) or atypical hepatic observations at contrast-enhanced CT: A prospective comparison. J Magn Reson Imaging, 2019, 49(2): 574-587.http://dx.doi.org/10.1002/jmri.26199

[38] Nakao S, Tanabe M, Okada M, Furukawa M, Iida E, Miyoshi K, et al. Liver imaging reporting and data system (LI-RADS) v2018: comparison between computed tomography and gadoxetic acid-enhanced magnetic resonance imaging. Jpn J Radiol, 2019, 37(9): 651-659.http://dx.doi.org/10.1007/s11604-019-00855-x

[39] Chen M F, Ho M C, Kao J H, Hwang R M, Deng S B, Yen K C, et al. Comparison of CT and gadoxetic acid-enhanced MRI with liver imaging reporting and data system to assess liver tumors before resection. J Formos Med Assoc, 2024, 123(3): 318-324.http://dx.doi.org/10.1016/j.jfma.2023.11.008

[40] Kim Y Y, Kim Y K, Min J H, Cha D I, Kim J M, Choi G S, et al. Intraindividual Comparison of Hepatocellular Carcinoma Washout between MRIs with Hepatobiliary and Extracellular Contrast Agents. Korean J Radiol, 2021, 22(5): 725-734.http://dx.doi.org/10.3348/kjr.2020.1143
[41] Yoon J K, Lee S, Hwang J A, Lee J E, Kim S S, Kim M J. Diagnostic performance of the 2022 KLCA-NCC criteria for hepatocellular carcinoma on magnetic resonance imaging with extracellular contrast and hepatobiliary agents: comparison with the 2018 KLCA-NCC criteria. J Liver Cancer, 2023, 23(1): 157-165.http://dx.doi.org/10.17998/jlc.2023.02.07
